# Ethylene mediates the branching of the jasmonate‐induced flavonoid biosynthesis pathway by suppressing anthocyanin biosynthesis in red Chinese pear fruits

**DOI:** 10.1111/pbi.13287

**Published:** 2019-11-19

**Authors:** Junbei Ni, Yuan Zhao, Ruiyan Tao, Lei Yin, Ling Gao, Åke Strid, Minjie Qian, Juncai Li, Yuanjun Li, Jiaqi Shen, Yuanwen Teng, Songling Bai

**Affiliations:** ^1^ Department of Horticulture Zhejiang University Hangzhou Zhejiang China; ^2^ Zhejiang Provincial Key Laboratory of Integrative Biology of Horticultural Plants Hangzhou Zhejiang China; ^3^ The Key Laboratory of Horticultural Plant Growth Development and Quality Improvement the Ministry of Agriculture of China Hangzhou Zhejiang China; ^4^ ACON Biotech (Hangzhou) Co., Ltd Hangzhou Zhejiang China; ^5^ School of Science and Technology Örebro University Örebro Sweden; ^6^ Liaoning Province Institute of Pomology Xiongyue Liaoning China; ^7^ Yantai Academy of Agricultural Sciences Yantai Shandong China

**Keywords:** anthocyanin, ethylene, flavone, isoflavone, jasmonate, pear, transcriptome

## Abstract

Flavonoid accumulation in most fruits is enhanced by ethylene and jasmonate. However, little is known about the hormone functions related to red pear fruit coloration or their combined effects and potential underlying mechanisms. Various treatments were used to investigate the flavonoid metabolite profile and pear transcriptome to verify the effects of ethylene and jasmonate on flavonoid biosynthesis in red pear fruits as well as the mechanism behind this. Ethylene inhibits anthocyanin biosynthesis in red Chinese pear fruits, whereas jasmonate increases anthocyanin and flavone/isoflavone biosyntheses. The branching of the jasmonate‐induced flavonoid biosynthesis pathway is determined by ethylene. Co‐expression network and Mfuzz analyses revealed 4,368 candidate transcripts. Additionally, ethylene suppresses *PpMYB10* and *PpMYB114* expression via TF repressors, ultimately decreasing anthocyanin biosynthesis. Jasmonate induces anthocyanin accumulation through transcriptional or post‐translational regulation of TFs‐like MYB and bHLH in the absence of ethylene. However, jasmonate induces ethylene biosynthesis and the associated signalling pathway in pear, thereby decreasing anthocyanin production, increasing the availability of the precursors for flavone/isoflavone biosynthesis and enhancing deep yellow fruit coloration. We herein present new phenotypes and fruit coloration regulatory patterns controlled by jasmonate and ethylene, and confirm that the regulation of fruit coloration is complex.

## Introduction

Flavonoids, which are vital secondary metabolites in plants, are determinants of fruit quality and commercial value because they influence fruit colour, nutritive value, aroma and antioxidant properties. The various classes of flavonoids, such as anthocyanins, flavones, isoflavones, proanthocyanins (PAs) and flavonols, are synthesized via the phenylpropanoid pathway (Shirley, 2001). Early flavonoid biosynthesis pathway genes (EBGs), such as *PAL* (phenylalanine ammonia lyase), *CHS* (chalcone synthase), *CHI* (chalcone isomerase) and *F3*′*H* (flavanone 3′‐hydroxylase), are involved in the production of common precursors. The downstream genes, namely the late biosynthetic genes (LBGs), are specifically expressed for various flavonoid derivatives. For example, *DFR* (dihydroflavonol 4‐reductase), *ANS* (anthocyanin synthase) and *UFGT* (UDP‐glucose:flavonoid 3‐glucosyltransferase) are the LBGs for anthocyanin biosynthesis, whereas *DFR*, *ANS*, *LAR* (leucoanthocyanidin reductase) and *ANR* (anthocyanin reductase) are the LBGs for PA biosynthesis. Flavones and isoflavones are synthesized at a branch point of the anthocyanin/PA pathway, with flavanones serving as the direct precursors. Additionally, *FNS* (flavone synthase) is the key flavone biosynthetic gene, and *IFS* (isoflavone synthase) as well as *IFD* (isoflavone dehydratase) is the key isoflavone biosynthetic genes. Moreover, *FLS* (flavonol synthase) and *UFGT* are the LBGs involved in flavonol biosynthesis (Martens and Mithöfer, 2005; Shirley, 2001). In diverse higher plant species, the flavonoid biosynthesis pathway is transcriptionally regulated by a conserved MYB‐bHLH‐WD40 (MBW) complex comprising a MYB transcription factor (TF), a basic helix‐loop‐helix (bHLH) and a WD‐repeat protein (Shirley, 2001; Xu *et al.*, 2015; Zoratti *et al.*, 2014). Several R2R3‐MYB family members separately control flavonoid biosynthesis through various flavonoid pathway branches (Dubos *et al.*, 2010; Jaakola, 2013).

Flavonoid biosynthesis is also controlled by diverse internal and external factors, of which plant hormones function as crucial regulators. Ethylene plays a key role during the ripening of many fruit species (Giovannoni, 2004). Additionally, previous studies revealed that ethylene can induce the biosynthesis of flavonoids, including anthocyanins, in the fruits of rosaceous plant species, such as apple (An *et al.*, 2018; Faragher and Brohier, 1984; Whale and Singh, 2007), strawberry (Given *et al.*, 1988) and plum (Cheng *et al.*, 2016). However, ethylene also negatively affects anthocyanin biosynthesis in some species. For example, an ethylene treatment after the lag phase in *Sorghum vulgare* inhibits anthocyanin accumulation (Craker *et al.*, 1971). Transgenic tobacco plants carrying the mutated melon ethylene receptor gene *CmETR1/H69A* are less sensitive to ethylene and have a higher anthocyanin level than wildtype plants (Takada *et al.*, 2005). In *Arabidopsis thaliana*, ethylene suppresses anthocyanin accumulation by suppressing the expression of TF genes encoding positive regulators (e.g. *GL3*, *TT8* and *PAP1*), while inducing the expression of TF genes encoding negative regulators (e.g. the R3‐MYB gene *MYBL2*). Thus, the ethylene‐mediated inhibition of anthocyanin accumulation appears to involve the ethylene signalling pathway. This is supported by the reported observation that anthocyanin levels are lower than normal in the ethylene insensitive mutants*etr1‐1*, *ein2‐1* and the *ein3 eil1* double mutant (Jeong *et al.*, 2010). However, little is known regarding the molecular mechanisms underlying the inhibition of anthocyanin biosynthesis by ethylene.

Jasmonate induces flavonoid accumulation in many fruits, such as apple (Rudell *et al.*, 2002), red raspberry (Flores and Ruiz del Castillo, 2014) and blackberry (Wang *et al.*, 2008). In apple, jasmonate ZIM‐domain (JAZ) proteins, which are the substrates of the SCF^COI1^ complex, interact with MdbHLH3 in the MBW complex to inhibit its function, thereby preventing the formation and transcriptional activity of the MBW complex. Jasmonate induces the degradation of JAZ proteins to release MdbHLH3, which promotes anthocyanin accumulation (An *et al.*, 2015). A very similar phenomenon occurs in *A. thaliana* (Qi *et al.*, 2011). Thus, the regulatory mechanism of jasmonate‐induced flavonoid biosynthesis appears to be conserved among plant species. However, previous studies of jasmonate‐regulated flavonoid biosynthesis were mainly focused on anthocyanin and PA biosyntheses. Additionally, jasmonate and ethylene synergistically promote anthocyanin biosynthesis in ‘Fuji’ apple fruits (Rudell and Mattheis, 2008). However, it remains unknown whether this synergistic effect also occurs in other fruit species.

Pears (*Pyrus* spp.) are important temperate fruit species that belong to the subtribe Malinae of the tribe Maleae in the family Rosaceae (Potter *et al.*, 2007). Pear and apple belong to the same subtribe and have highly similar genomes (Wu *et al.*, 2013). In Asian pear fruits, PpMYB10 and PpMYB114 regulate anthocyanin biosynthesis (Feng *et al.*, 2010; Yao *et al.*, 2017). Moreover, PpMYB9 induces anthocyanin, flavonol and PA biosyntheses by activating the *PpANR* and *PpUFGT1* promoters (Zhai *et al.*, 2016). However, the functions of ethylene related to red pear fruit coloration have not been comprehensively characterized. Furthermore, the combined effects of jasmonate and ethylene on red pear fruit coloration remain unclear.

In this study, we observed that ethylene treatment markedly inhibited red pear fruit coloration, which is inconsistent with the effect of ethylene in other fruits (e.g. apple). Furthermore, ethylene and jasmonate functioned antagonistically and synergistically during the regulation of anthocyanin biosynthesis and flavone/isoflavone biosynthesis, respectively. To clarify the potential regulatory network, we improved the annotation of a red pear genome and generated new information regarding a reference transcriptome database via Pacific Biosciences (PacBio) full‐length (FL) sequencing and RNA sequencing (RNA‐seq). By constructing co‐expression networks and analysing transcript expression trends, we identified candidate transcripts differentially responsive to ethylene and jasmonate and further analysed the potential regulatory patterns of the structural gene transcripts involved in flavonoid biosynthesis. Our results identified ethylene‐ and jasmonate‐responsive transcripts, which are correlated with anthocyanin biosynthesis or the deep yellow coloration of fruits. These findings would be useful for clarifying the mechanism underlying ethylene‐ and jasmonate‐regulated flavonoid biosynthesis. Our data also provide new insights into the regulatory effects of jasmonate and ethylene on the coloration of fruit tree crops.

## Results

### Effects of ethylene and jasmonate on the red coloration of pears and apples

**Figure 1:**
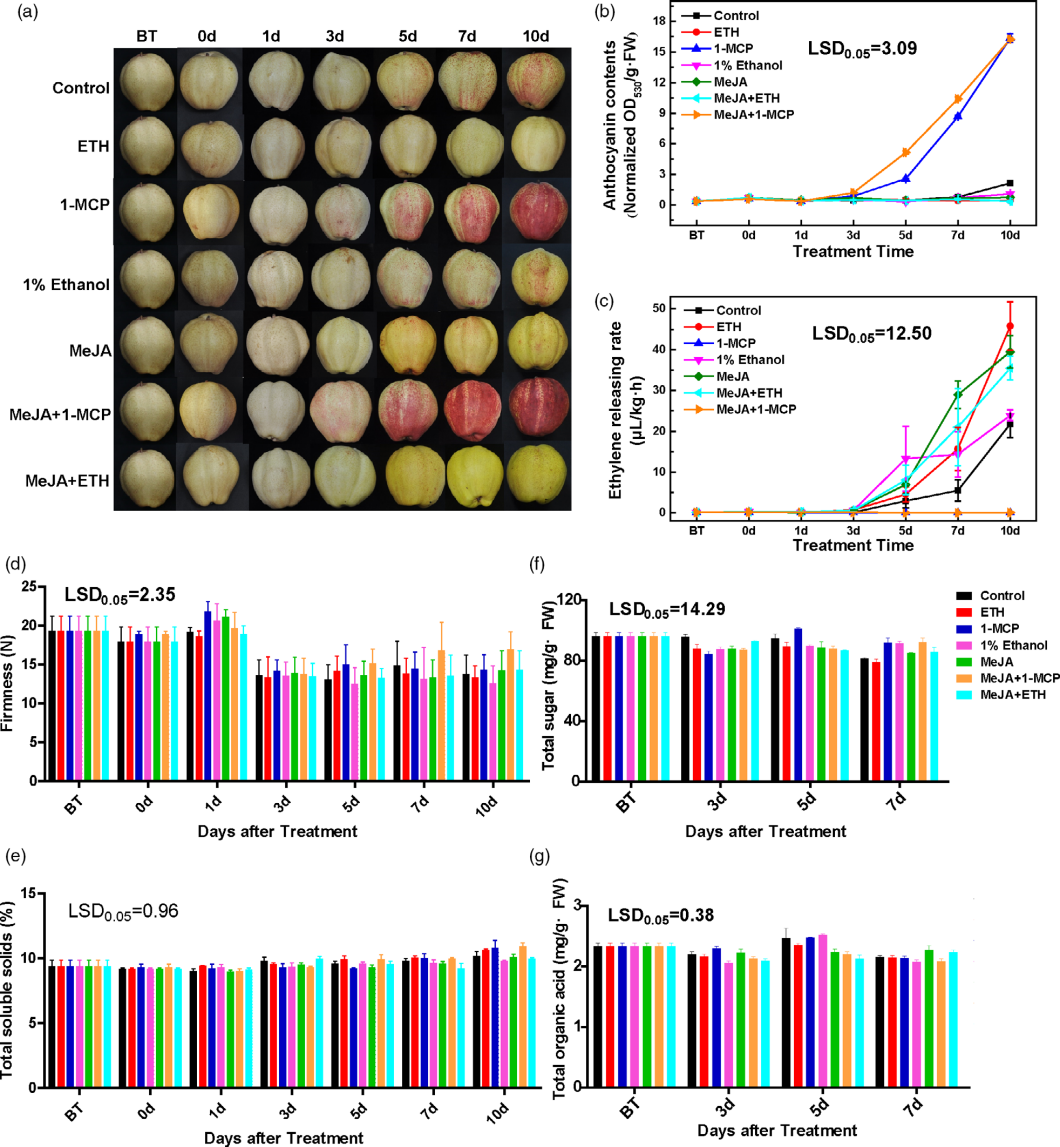
Effects of various treatments on the ‘Hongzaosu’ pear fruit coloration. (a) Representative images of ‘Hongzaosu’ pear fruits following diverse treatments. (b–g) Effects of various treatments on the total anthocyanin content (b), ethylene‐release rate (c), fruit firmness (d), total soluble solid content (e), total sugar content (f) and total organic acid content (g). ETH, fruit treated with ethephon; MeJA, fruit treated with methyl jasmonate; 1‐MCP, fruit treated with 1‐methylcyclopropene. Data are presented as the mean ± standard error of three biological replicates.

The red coloration of the ‘Hongzaosu’ control fruits was first observed at 5 days after initiating the light treatment. Thereafter, the red coloration continued to intensify (Figure 1a). Pear fruits treated with ethephon (ETH) exhibited no obvious changes, whereas the 1‐MCP treatment obviously induced the red coloration at 3 days after initiating the light treatment (Figure 1a). The red coloration was accompanied by increased anthocyanin accumulation (Figure 1b). An analysis of ethylene production revealed that 1‐MCP prevented the release of ethylene. In contrast, the ETH treatment induced ethylene production (Figure 1c). These results indicated that ethylene inhibited light‐induced anthocyanin biosynthesis in the red pear fruits.

The MeJA treatment induced a deep yellow coloration of ‘Hongzaosu’ pear fruits (Figure 1a, Figure S2). A similar phenotype was observed in response to the MeJA + ETH treatment. Additionally, the MeJA and MeJA + ETH treatments significantly induced ethylene production, similar to the ETH treatment (Figure 1c). Thus, the changes induced by the MeJA treatment were due to the combined effects of jasmonate and ethylene. However, the MeJA + 1‐MCP treatment inhibited ethylene production (Figure 1c), resulting in red coloration and anthocyanin accumulation (Figure 1a,b). The MeJA + 1‐MCP treatment did not induce the yellow coloration of fruits (Figure S2). These results suggested that jasmonate induced the red coloration and anthocyanin biosynthesis of fruits in the absence of ethylene, but induced the deep yellow coloration in the presence of ethylene.

We also examined the changes in fruit firmness, total soluble solid (TSS) content and the sugar/acid concentrations of the fruit flesh during treatments (Figure 1d–g, Figure S3). The fruit firmness of all samples significantly decreased at 3 days after treatments, whereas the TSS content was minimally affected. Although the composition of sugars and acids varied slightly among the treatments at some time points, the changes in the total sugars and acids induced by the treatments were not significant during the treatment period, which is consistent with the tendency of the TSS content. These results suggest that the treatments (especially with ethylene) affected the pigmentation of the ‘Hongzaosu’ pear fruit, but had few effects on fruit quality and the ripening process.

To exclude cultivar‐specific effects of ethylene and MeJA, we analysed fruits from other red pear cultivars with diverse genetic backgrounds. ‘Mantianhong' pear (*Pyrus pyrifolia*), ‘Nanhong' pear (*Pyrus ussuriensis*) and ‘Red Clapp's Favorite' pear (*Pyrus communis*) had similar responses (Figures S4, S5 and S6). We also treated ‘Fuji'apple fruits to test the reliability of the treatment methods. For apple, we obtained the opposite results compared with those for pear fruits. The 1‐MCP treatment inhibited the red coloration of apple fruits, whereas the ETH treatment induced it (Figure S7). Moreover, MeJA and MeJA + ETH treatments also induced the red coloration, whereas MeJA + 1‐MCP inhibited the red coloration. Yellow coloration of fruits was not observed, regardless of treatment.

### Carotenoid and flavonoid profiling

To identify the pigment compounds responsible for the deep yellow coloration after the MeJA treatment, carotenoid and flavonoid contents were measured. The total carotenoid contents induced by 1‐MCP, MeJA, MeJA + ETH and MeJA + 1‐MCP treatments were inconsistent with the observed phenotypes (Figure 1a and Figure S8A). Furthermore, the control, 1‐MCP‐treated and MeJA‐treated samples were screened for 18 types of carotenoid compounds by LC‐MS/MS; only nine carotenoids were detected (Figure S8B), but their amounts were inconsistent with the phenotypes (Figure S8B and Figure 1). Furthermore, only violaxanthin and zeaxanthin contents were higher after the MeJA treatment than after the 1‐MCP treatment. The concentrations of these compounds were <45 μg/g and 15 μg/g, respectively (Figure S8B). These results suggested that carotenoids were not the dominant pigments responsible for the deep yellow coloration, but they may function as copigments (Martens and Mithöfer, 2005; Tanaka *et al.*, 2008).

**Figure 2:**
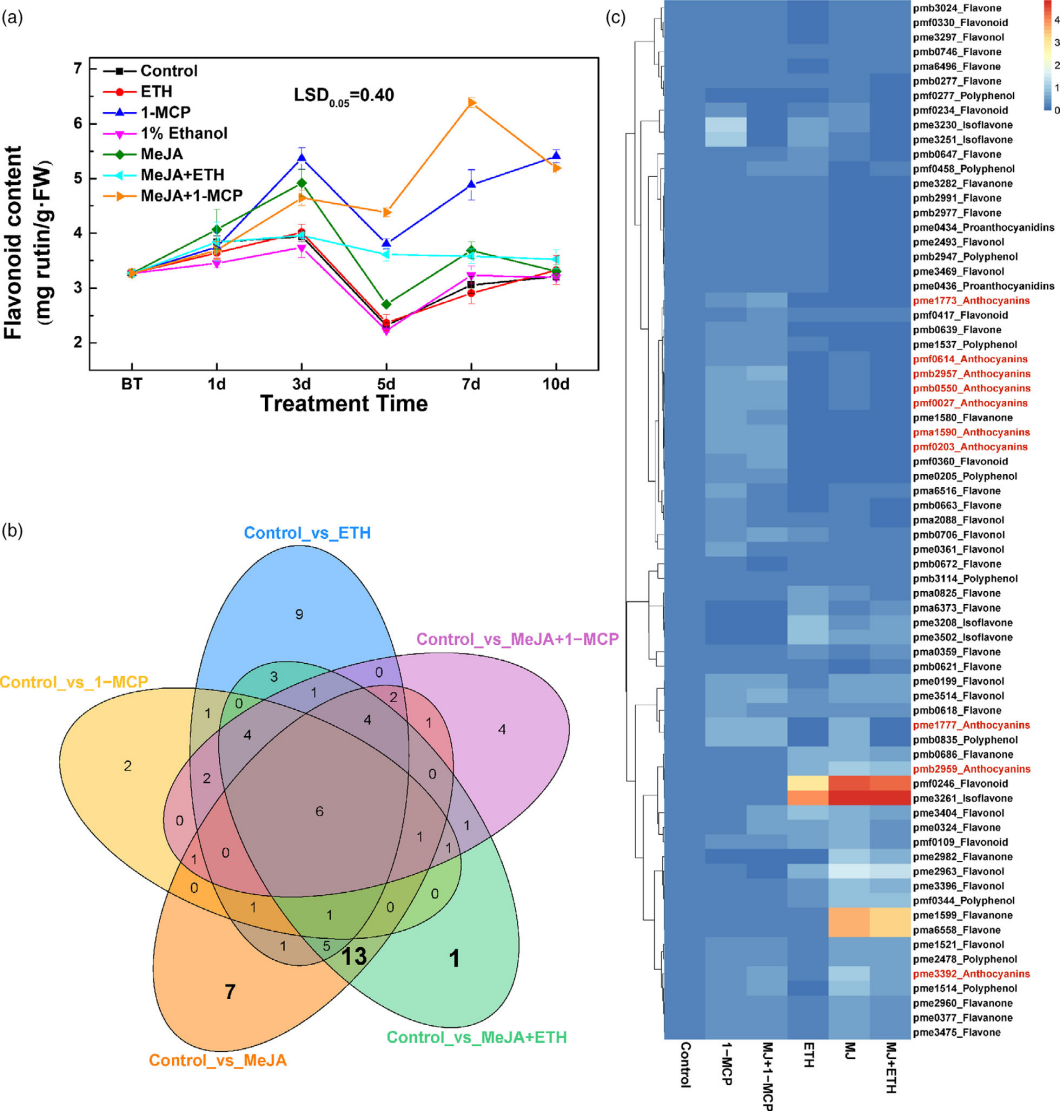
Effects of various treatments on flavonoid biosynthesis in ‘Hongzaosu’ pear fruits. (a) Total flavonoid contents of ‘Hongzaosu’ pear fruits following diverse treatments. (b) Venn diagram of differentially accumulated flavonoid metabolites in each sample pair. (c) Heat map indicating the relative flavonoid contents. The corresponding flavonoids for the index are listed in Table S2. Data are presented as the mean ± standard error of three biological replicates.

In contrast, the flavonoid contents were highly related with the red and yellow coloration. The MeJA + 1‐MCP and 1‐MCP treatments significantly increased the total flavonoid content, likely because of the substantial accumulation of anthocyanin (Figures 1b and 2a). Moreover, compared with control levels, a greater abundance of flavonoids was observed at 3–7 days after the MeJA and MeJA + ETH treatments, which induced the yellow coloration (Figures 1a and 2a). Therefore, the yellow coloration induced by MeJA and MeJA + ETH was mainly due to the accumulation of flavonoid compounds (excluding anthocyanin).

To analyse the red and yellow pigments in detail, we profiled the flavonoid metabolites resulting from the control, ETH, 1‐MCP, MeJA, MeJA + ETH and MeJA + 1‐MCP treatments. A principal component analysis revealed that the main flavonoid categories differed between the treated and control samples (Figure S9). Of 211 tested flavonoid compounds, 72 differentially accumulated metabolites were detected and analysed further (Figure 2b,c and Table S2), of which the abundance of nine anthocyanins was highly increased by 1‐MCP and MeJA + 1‐MCP treatments (Figure 2c). Additionally, 21‐specific flavonoids (Figure 2b and Table S2), including six flavones, four flavonols, four flavanones, five polyphenols and two PAs (Table S2), were identified following the MeJA and MeJA + ETH treatments. Additionally, the MeJA and MeJA + ETH treatments increased the contents of pme3261 (isoflavone, 4',6,7‐trihydroxyisoflavone), pmf0246 (flavonoid, luteolin‐8‐C‐glucoside), pma6558 (flavone, velutin) and pme1599 (flavanone, 7‐methoxy‐5,3',4'‐trihydroxyflavanone) by more than 4,000‐fold compared with the levels induced by the control, 1‐MCP and MeJA + 1‐MCP treatments (Figure 2c, Table S2). These results indicated that flavones and isoflavones may be responsible for the deep yellow coloration induced by the MeJA and MeJA + ETH treatments.

### PacBio sequencing and construction of the ‘Hongzaosu’ pear transcriptome

Considering the large difference between the published pear genome and the ‘Hongzaosu’ pear genome, we applied PacBio technology to sequence the ‘Hongzaosu’ pear transcriptome. Three size‐fractionated libraries (1–2, 2–3 and 3–6 kb) were constructed to avoid loading bias (Figure S10). The PacBio sequencing identified 75 812 isoforms, including 58 513 polished high‐quality isoforms and 16 669 polished low‐quality isoforms (Table S3), which were further revised with Illumina RNA‐seq data. These consensus isoforms were then mapped to the ‘Suli’ pear genome and collapsed into 28 321 nonredundant isoforms (Figure S11A). The average length of these isoforms was 2073 bp, which was much longer than the isoforms in the reference pear genome (948 bp) (Figure S11B). We then compared the 28 321 nonredundant isoforms against the 42 369 transcripts in the *Pyrus bretschneideri* Rehd. pear genome dataset (V121010) (Figure S11A). Among the 28 321 nonredundant isoforms, 6215 (21.94%) were mapped to the genome dataset, and 20 458 (72.24%) were new isoforms of annotated genes. Additionally, 1648 isoforms (5.82%) were from 1180 novel loci that were not annotated in the ‘Suli’ genome. To clarify the origin and coding potential of these novel loci, we aligned these isoforms to annotated gene and protein sequences of the genetically related species apple (*Malus domestica*) and peach (*Prunus persica*) (BLASTX; e‐value ≤ 1e‐10). A total of 1273 isoforms (77.25%) were present in both genomes, whereas 179 (10.85%) exhibited homology to sequences in one of the genomes. The remaining isoforms (196) were not homologous to any sequences in either genome, suggesting they are species specific.

**Figure 3:**
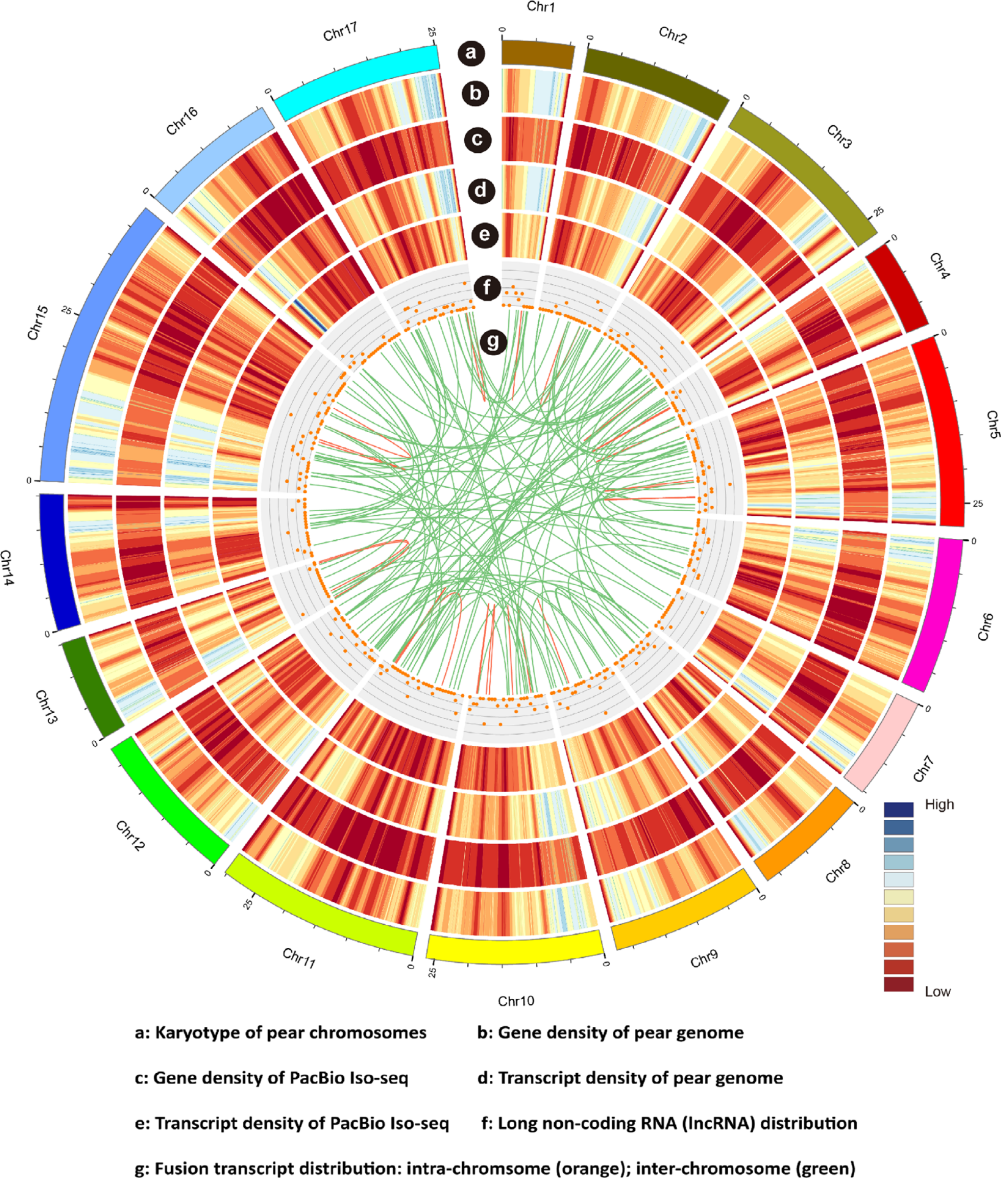
CIRCOS visualization of data at the genome‐wide level. (a) Karyotype of pear chromosomes. (b) Gene density for the pear genome. (c) Gene density for Pacific Biosciences (PacBio) Iso‐seq. (d) Transcript density for the pear genome. (e) Transcript density for PacBio Iso‐seq. (f) Long noncoding RNA (lncRNA) distribution in 1 Mb bins on each chromosome. (g) Fusion transcript distribution: intra‐chromosome (orange) and inter‐chromosome (green).

Compared with the annotated pear genome dataset (V121010), in which only one isoform of each locus was annotated (Figure 3a,b,d), our PacBio dataset included 28 321 transcripts from 16 064 loci, with an average of 1.76 isoforms per loci (Figure 3a,c,e). Among the five main types of alternative splicing events, intron retention was the most common, accounting for 57.81% of all alternative splicing events, followed by alternative 3′ splicing (20.61%), whereas mutually exclusive exons (1.22%) were the least common (Figure S11C). The locus with the most isoforms (43) was on chromosome 8 (10 660 636–10 668 780) (Gene ID: Pbr018758.1) (Figure S12) and encoded an L‐arabinokinase. To further confirm the high fidelity of the long‐read sequencing strategy, we validated the expression of three genes by RT‐PCR. We observed that different isoforms were differentially expressed during coloration (Figure S13).

Finally, we combined the 1648 transcripts from novel loci and the 20 458 novel isoforms from annotated loci with the annotated pear genome gene dataset (42 369 transcripts) to generate a comprehensive pear reference sequence database containing 64 775 transcripts from 43 549 loci. This reconstructed transcript dataset provided precise and detailed information regarding sequences and alternative splicing, and was used as a reference dataset for our subsequent analyses.

### RNA‐seq data revealed differentially expressed genes during ‘Hongzaosu’ fruit coloration

The RNA‐seq analysis of fruit peel samples generated 412.70 Gb of clean data (Table S4). The above‐mentioned transcript dataset was used to identify differentially expressed transcripts (DETs). A pairwise comparison of each sample detected 18 538 DETs (Figure S14). Six randomly selected DETs were analysed by qRT‐PCR, which indicated the expression levels were consistent with the RNA‐seq data (Figure S15), thus confirming the accuracy and reliability of the RNA‐seq results.

### Analysis of DET expression trends

**Figure 4:**
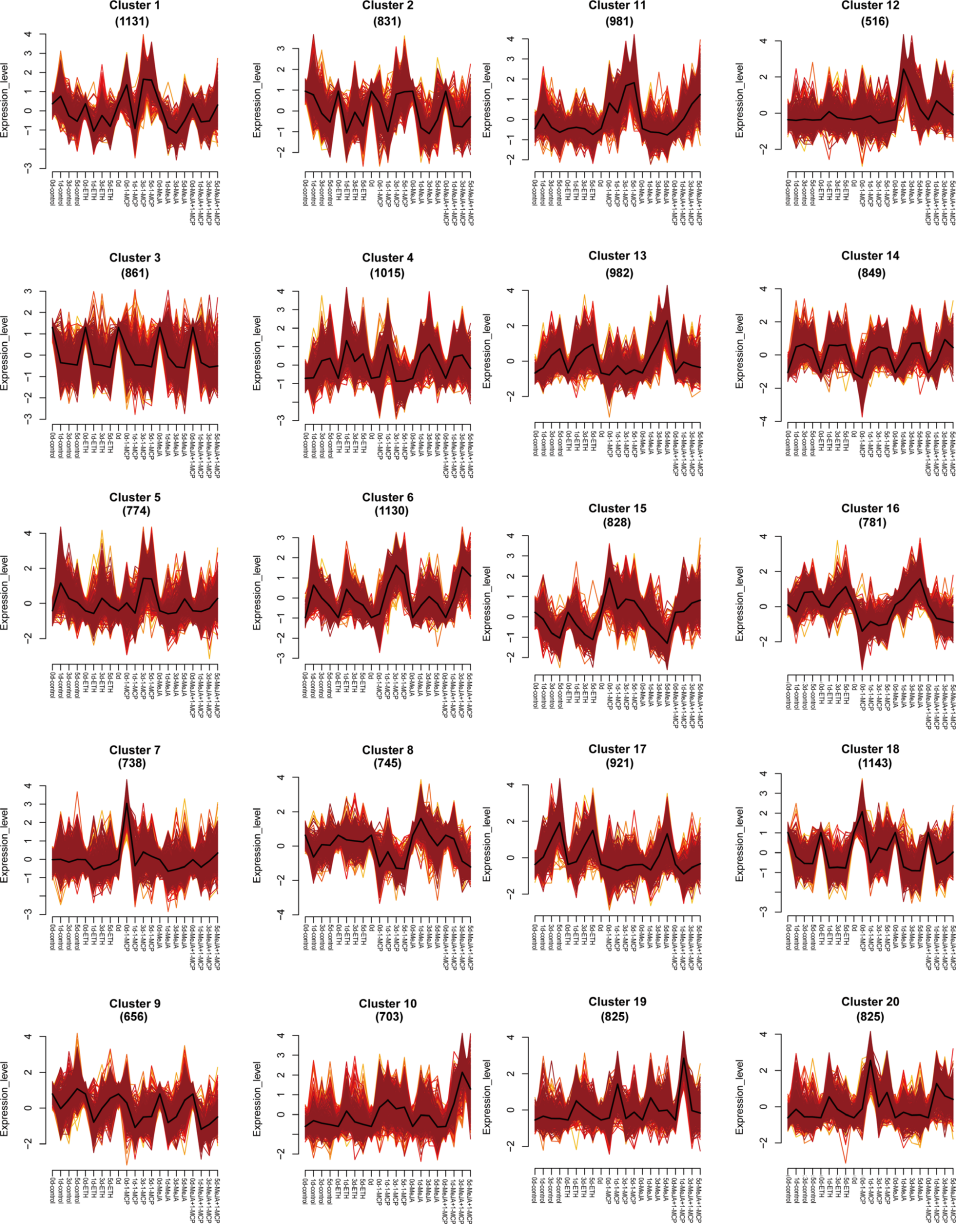
Results of the Mfuzz clustering of 17 235 differentially expressed transcripts identified by Pacific Biosciences (PacBio) Iso‐Seq based on their expression patterns.

To investigate how the DETs responded to jasmonate or ethylene signalling, 17 235 DETs (92.97%) were grouped into 20 clusters by Mfuzz based on their expression levels (Figure 4 and Table S5). Transcripts from clusters 3, 9 and 14 showed similar expression tendency after different treatments, and transcripts from clusters 1, 2 and 4 showed no obvious regular pattern to different treatments (Figure 4). Expression levels of DETs from clusters 5, 6, 11 and 20 were gradually induced by 1‐MCP treatment, while expression levels of DETs from clusters 7, 15 and 18 were rapidly up‐regulated after 1‐MCP treatment. But expressions of all these seven clusters showed no significant change after MeJA + 1‐MCP treatment compared with 1‐MCP treatment (Figure 4). These results indicated that transcripts in these seven clusters were negatively responsive to ethylene. Furthermore, DETs from cluster 17 showed very low expression levels after 1‐MCP and MeJA + 1‐MCP treatment and showed no obvious difference between ETH treatment and MeJA treatment (Figure 4), indicating that transcripts from cluster 17 positively responded to ethylene. Expression of DETs from clusters 10 and 19 showed higher expression level after MeJA + 1‐MCP treatment than that after 1‐MCP treatment (Figure 4), indicating that these DETs positively responded to jasmonate and negatively responded to ethylene. Moreover, expression levels of DETs from clusters 8, 13 and 16 were highly induced by MeJA and ETH treatment and inhibited by 1‐MCP and MeJA + 1‐MCP treatment (Figure 4). These results suggested that these DETs positively responded to both jasmonate and ethylene, and ethylene played dominant roles, because 1‐MCP inhibited the induction effect of MeJA. DETs from cluster 12 showed highly increased expression levels after MeJA treatment, and this induction effect was partly inhibited by 1‐MCP treatment (Figure 4). These results suggested that DETs from cluster 12 positively responded to jasmonate and ethylene, but jasmonate played dominant roles. Thus, we classified the DETs into eight groups (Table 1) according to their expression tendencies after diverse treatments. The DETs from groups 3–8 were potentially involved in the jasmonate‐ and/or ethylene‐regulated coloration. To further analyse the potential function of these DETs, we completed a KEGG pathway enrichment analysis of groups 3–8 (Figure S16). Most of the DETs in these six groups were related to hormone signal transduction, flavonoid biosynthesis or phenylpropanoid biosynthesis (Figure S16), implying that our method for selecting candidate DETs was reliable.

**Table 1:** Hormone signal responsive category analysis of DETs based on Mfuzz cluster analysis

Group	Clusters	Response	Numbers of DETs
Group1	2, 3, 9, 14	No response	3197
Group2	1, 4	No obvious change pattern	2146
Group3	5, 6, 11, 20	Negatively respond to ethylene	3710
Group4	7, 15, 18	Rapidly negatively respond to ethylene	2709
Group5	17	Positively respond to ethylene	921
Group6	10, 19	Positively respond to jasmonate and negatively respond to ethylene	1528
Group7	8, 13, 16	Positively respond to ethylene (dominant) and jasmonate	2508
Group8	12	Positively respond to ethylene and jasmonate (dominant)	516

### Differentially expressed transcripts involved in ethylene and jasmonate biosynthesis and signal transduction

**Figure 5:**
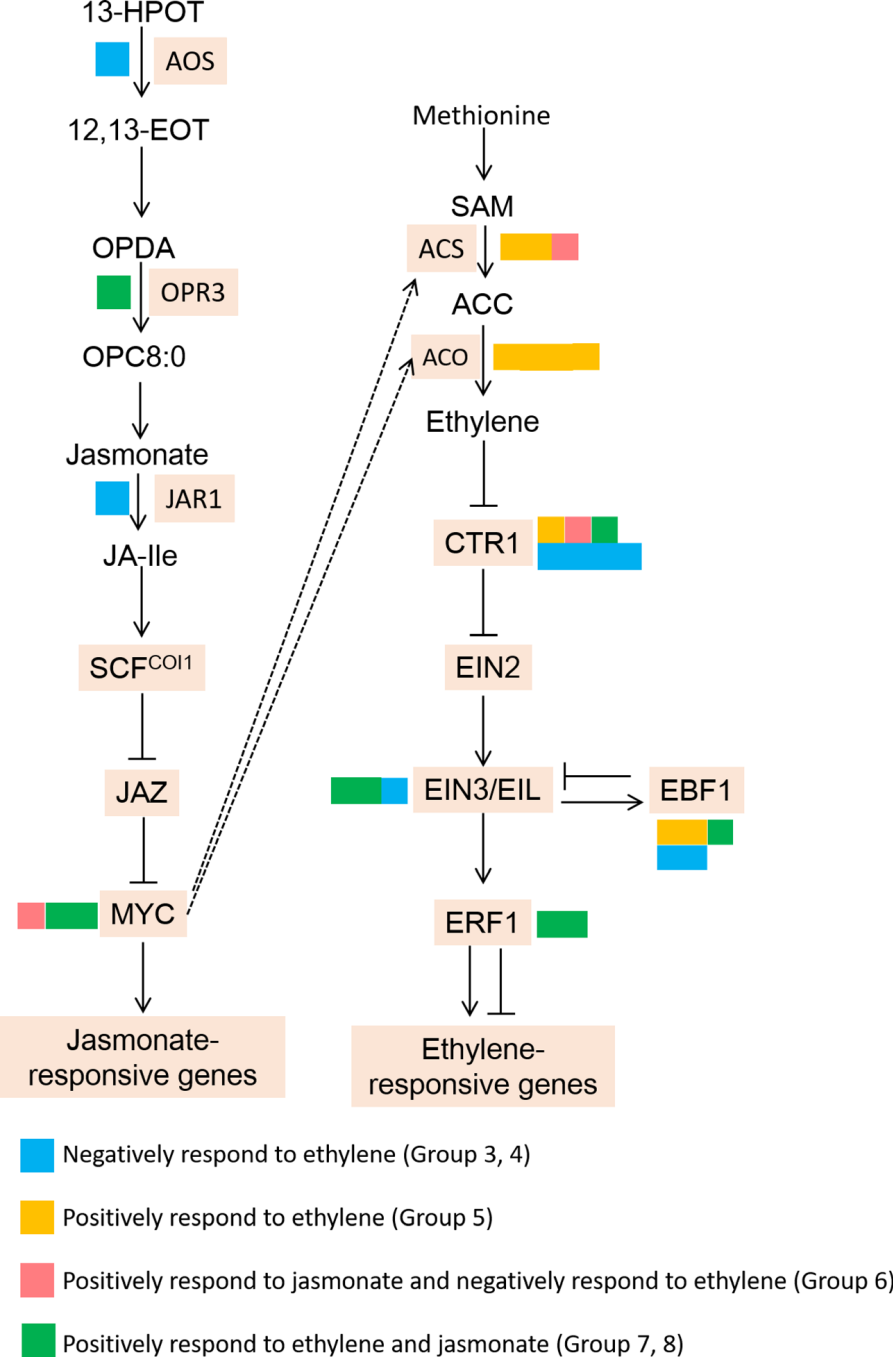
Categorization of genes belonging to jasmonate or ethylene biosynthesis or signalling transduction pathways. The genes in these pathways were assigned to different gene sets based on their responsiveness.

To determine the functions of ethylene and jasmonate during pear fruit coloration, the expression patterns of DETs involved in their biosynthesis and signal transduction pathways were analysed (Figure 5). The expression of key ethylene biosynthesis genes (*PpACS* and *PpACO* genes) increased in response to ethylene. The exception was one *PpACS* gene, for which ethylene suppressed expression, whereas jasmonate had the opposite effect. The *PpCTR1* genes, which encode negative regulators of ethylene, were classified into four groups, most of which were negatively responsive to ethylene. Moreover, the expression levels of most *PpEIN3/EIL* genes increased in response to ethylene and jasmonate, suggesting that jasmonate positively regulated the ethylene signalling pathway following our treatments. The *PpEBF1* genes, which contribute to the negative feedback control of *PpEIN3/EIL* expression, exhibited diverse response patterns to ethylene, indicative of the complexity of the ethylene signalling pathway. The *PpERF1*genes, which encode a downstream positive regulator of *PpEIN3/EIL* expression, positively responded to ethylene and jasmonate. Furthermore, the jasmonate biosynthesis pathway genes *Pp* *AOS* and *Pp* *JAR1* were negatively responsive to ethylene. However, *Pp* *OPR3* expression was induced by ethylene, implying the regulatory effects of ethylene on jasmonate biosynthesis are complex. The *PpMYC* genes, which are targeted by PpJAZs, positively responded to jasmonate, but exhibited variable responses to ethylene.

### Analyses of co‐expression networks revealed anthocyanin‐ and flavone/isoflavone‐related DETs

**Figure 6:**
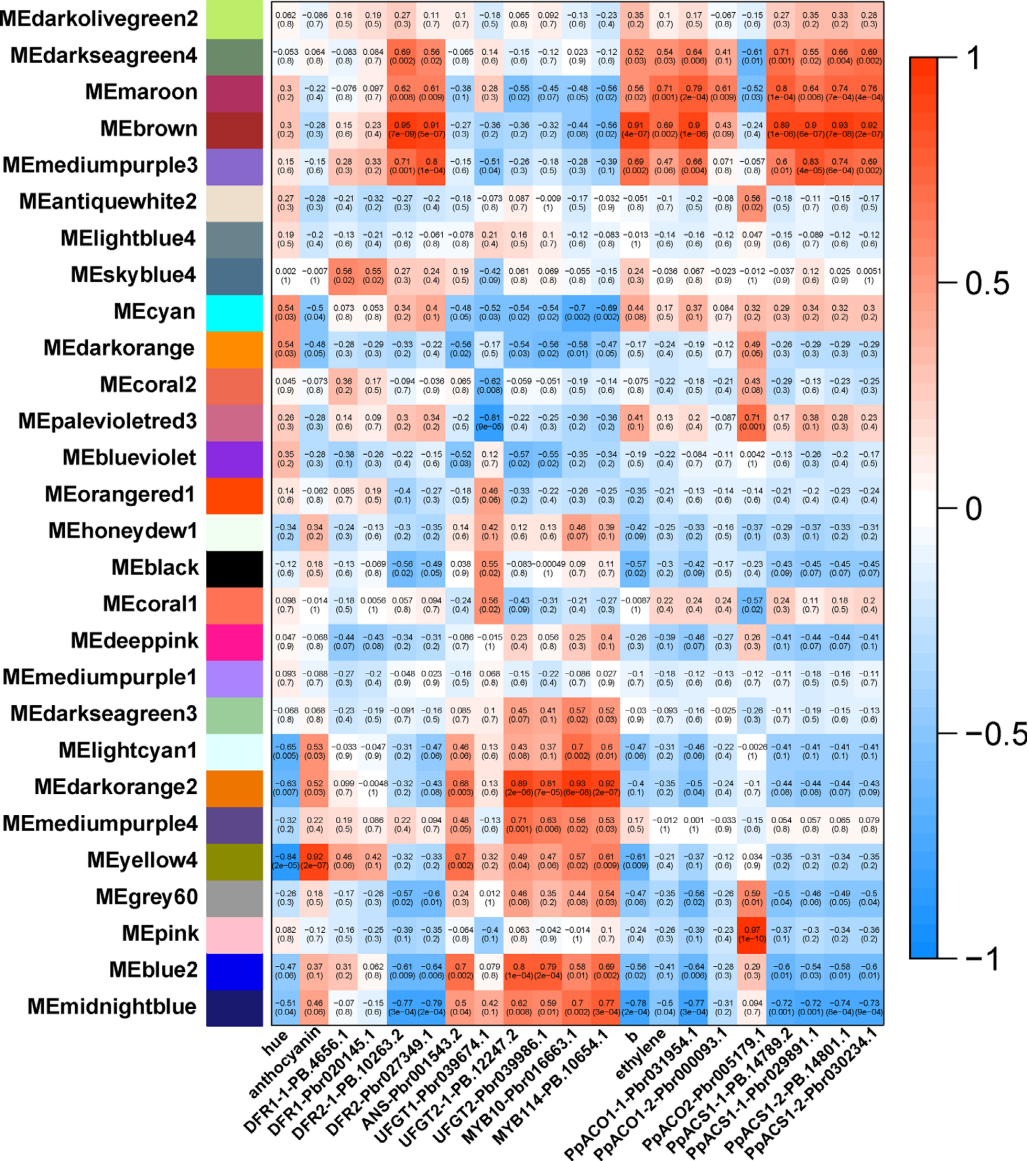
Identification of transcripts by the weighted gene co‐expression network analysis (WGCNA) of hormone‐treated ‘Hongzaosu’ pear fruit peels. Module–trait correlations and the corresponding *P*‐values are in parentheses. The left panel presents the 28 modules. The colour scale on the right presents the module–trait correlations from − 1 (blue) to 1 (red). The panels “hue”, “anthocyanin”, “b”, and “ethylene” represent the hue angles, anthocyanin contents, b* values and ethylene‐release rates, respectively. The other panels represent the changes in gene expression levels.

To identify anthocyanin biosynthesis‐related transcripts, we performed a weighted gene co‐expression network analysis (WGCNA) of all DETs, which identified 28 WGCNA modules (Figure 6 and Figure S17). The analysis of module–trait relationships revealed that the “yellow4” module was highly negatively correlated with the *hue* angle (*r *= −0.84, *P* = 2 × 10^‐5^) and highly positively correlated with anthocyanin content (*r* = 0.92, *P* = 2 × 10^‐7^) in the 51 samples. To further select DETs related to anthocyanin biosynthesis, a module–trait relationship analysis was performed using the RPKMs of *PpDFR*, *PpANS*, *PpUFGT*, *PpMYB10* and *PpMYB114* as the trait data. The results revealed that *PpANS* and *PpUFGT2* showed similar expression pattern to that of *PpMYB10* and *PpMYB114*, while *PpDFR2* showed opposite expression pattern of *PpMYB10* and *PpMYB114*. Furthermore, the analysis revealed that ‘lightcyan1’, ‘darkorange2’ and ‘midnightblue’ modules were highly positively correlated with the expression pattern of *PpMYB10* and *PpMYB114*, while ‘cyan’ module was highly negatively correlated with that. ‘Darkorange2’, ‘yellow4’, ‘mediumpurple4’ and ‘blue2’ modules were highly positively correlated with the expression pattern of *PpANS* and *PpUFGT2*. Based on the analysis above, we selected six modules positively correlated with anthocyanin biosynthesis (group 1) and one module negatively correlated with that (group 2) (Table 2) for further study.

**Table 2:** Statistics of module–traits with high correlations

Correlation	Related to anthocyanin biosynthesis		Related to flavone/isoflavone biosynthesis	
Positively	Group1	Yellow4 Lightcyan1 Darkorange2 Mediumpurple4 Blue2 Midnightblue	Group3	Brown Mediumpurple3
Negatively	Group2	Cyan	Group4	Midnightblue

‘Hongzaosu’ pear fruits began to turn deep yellow at 3 days after the MeJA and MeJA + ETH treatments (Figure 1). To identify transcripts related to deep yellow coloration, we performed a module–trait relationship analysis, with b* values as trait data (Figure 6). The ‘brown’ and “mediumpurple3” modules were highly positively correlated with the changes to the b* value (group 3), whereas the “midnightblue” module was highly negatively correlated (group 4) (Figure 6 and Table 2). Because the key structural genes (*PpFNSII*, *PpIFS* and *PpIFD*) and regulatory genes in the flavone/isoflavone biosynthesis pathway were not characterized in pear, we further analysed the three above‐mentioned modules (Table 2). We also performed a module–trait relationship analysis, with the ethylene‐release rate and the FPKMs of key ethylene biosynthesis genes (*PpACO* and *PpACS*) as the trait data. The co‐expression patterns were consistent with the b* value changes, except for *PpACO2* (Figure 6). Thus, ethylene may be vital for regulating flavone/isoflavone biosynthesis.

### Candidate DET set construction based on the Mfuzz and co‐expression analyses

The phenotypes as well as the physiological and biochemical evidence suggested that the following two types of DETs may influence ethylene‐inhibited anthocyanin biosynthesis: (1) DETs negatively responsive to ethylene signalling and positively correlated with anthocyanin biosynthesis; (2) DETs positively responsive to ethylene signalling and negatively correlated with anthocyanin biosynthesis. The following DET type may affect ethylene‐inhibited and jasmonate‐induced anthocyanin biosynthesis; (3) DETs negatively responsive to ethylene signalling, positively responsive to jasmonate signalling and positively correlated with anthocyanin biosynthesis. We combined the results of the Mfuzz and co‐expression analyses, and selected 2002, 540 and 502 DETs belonging to the above‐mentioned three categories, respectively (Table 3). Furthermore, we speculated that the following DET type may contribute to flavone/isoflavone biosynthesis co‐induced by ethylene and jasmonate; (4) DETs positively responsive to ethylene and jasmonate signalling and positively correlated with yellow coloration (b* value, Table 3 and Table S6). According to the Mfuzz and co‐expression analyses, 1324 DETs were selected (Table 3). Four candidate DET sets containing 4368 DETs were selected for further analyses (Table 3 and Table S6).

**Table 3:** Statistics of candidate transcripts sets

Sets				
Modules	Set 1	Set 2	Set 3	Set 4
	(‐) Ethylene (group 3, 4)	(+) Ethylene (group 5)	(+) Jasmonate, (‐) Ethylene (group 6)	(+) Jasmonate, (+) Ethylene (group 7, 8)
Positively related to anthocyanin biosynthesis (Group 1)	2002	–	502	–
Negatively related to anthocyanin biosynthesis (Group 2)	–	540	–	–
Positively related to flavone–isoflavone biosynthesis (Group 3)	–	–	–	1324
Total	4368			

### Flavonoid biosynthesis structural gene expression patterns

**Figure 7:**
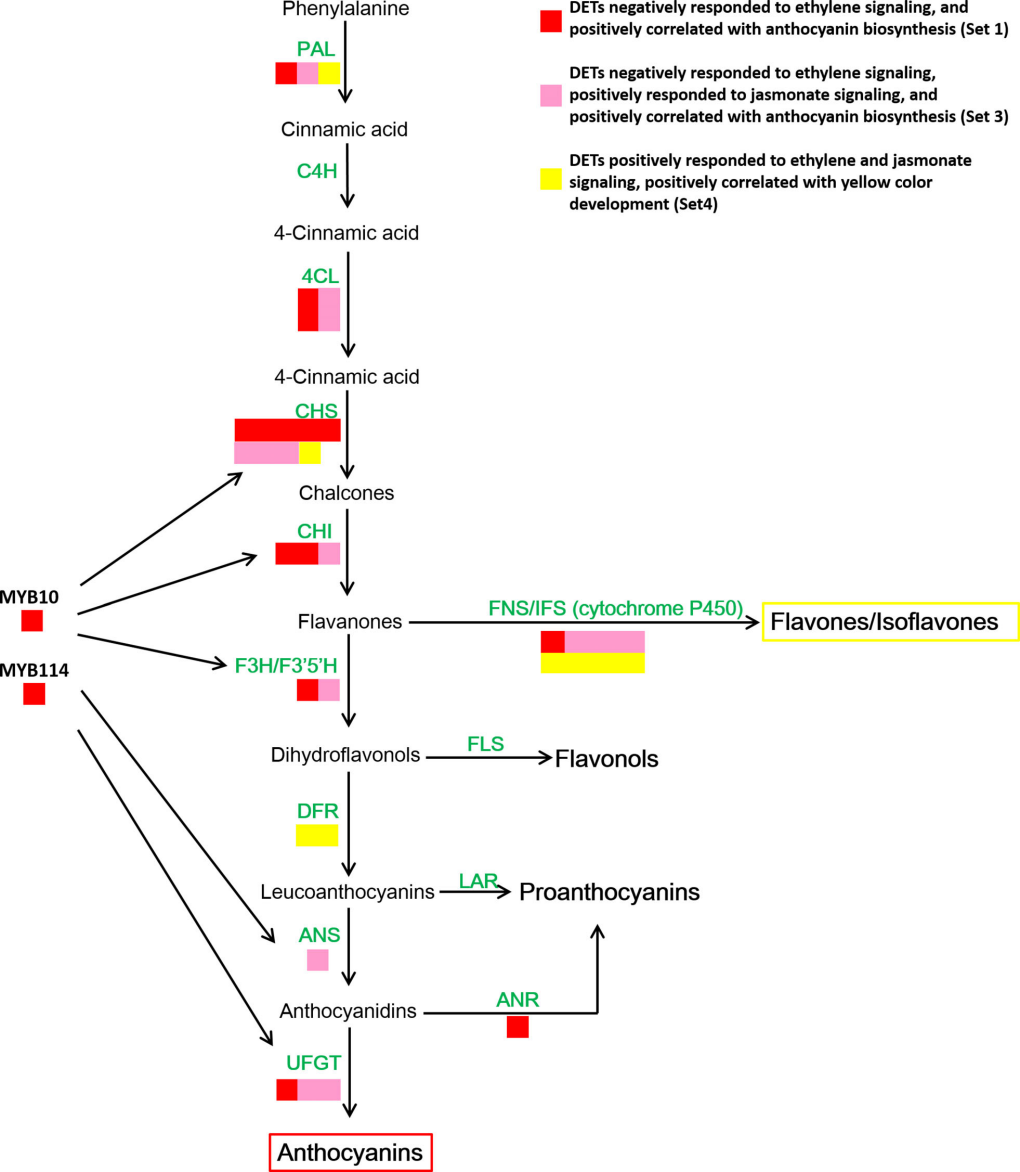
Categorization of genes belonging to the flavonoid biosynthesis pathway. Structural genes were assigned to different gene sets according to the co‐expression and Mfuzz analyses.

We mapped the selected anthocyanin‐ and flavone/isoflavone‐related genes to the flavonoid pathway to determine their expression patterns (Figure 7). Most anthocyanin EBGs, such as two *PpPAL*, four *Pp4CL*, eight *PpCHS* and three *PpCHI* genes, as well as the anthocyanin LBGs, such as *PpF3H*, *PpF3′5′H*, *PpANS* and *PpUFGT* genes, were positively related to anthocyanin biosynthesis and negatively responsive to ethylene and/or positively responsive to jasmonate (sets 1 and 3). Additionally, the expression levels of key anthocyanin biosynthesis regulatory genes (*PpMYB10* and *PpMYB114*) were positively correlated with anthocyanin accumulation, but were negatively responsive to ethylene. These results indicated that ethylene inhibited anthocyanin biosynthesis by suppressing the MBW complex via the transcriptional regulation of R2R3‐MYBs to down‐regulate the expression of some EBGs and most LBGs. However, all of the differentially expressed *PpDFR* genes were classified into Set 4, which was positively related to flavone/isoflavone biosynthesis, probably because these genes have nondominant roles during anthocyanin biosynthesis. The *PpANR* genes, which are important for PA biosynthesis, were negatively responsive to ethylene, implying ethylene inhibits PA production.

The *PpFNS* and *PpIFS* genes, which are key flavone/isoflavone biosynthesis genes, were predominantly classified into set 4 and were positively responsive to ethylene and jasmonate, as were one *PpPAL* and one *PpCHS* gene. Thus, jasmonate and ethylene‐induced flavone/isoflavone biosynthesis by promoting the expression of *PpFNS* and *PpIFS* genes and some EBGs. These results suggested that various EBGs are involved in diverse branches of the flavonoid biosynthesis pathway.

### Identification of transcription factor genes in candidate gene sets

**Figure 8:**
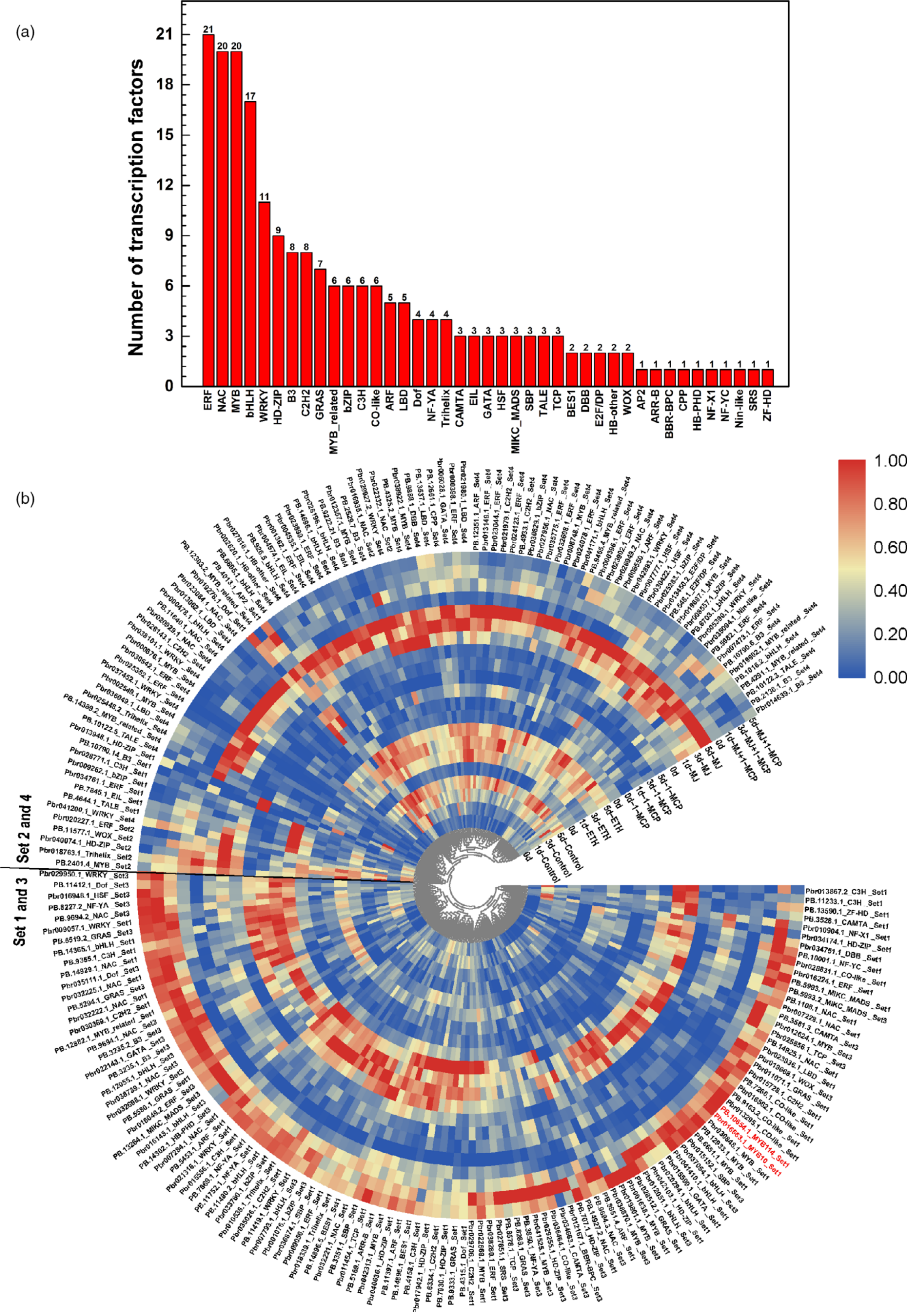
Transcription factors (TFs) involved in jasmonate‐ and ethylene‐regulated flavonoid biosynthesis. (a) Number of TFs in different families. (b) Heat map presenting the expression patterns of different TFs in response to diverse treatments. The heat map was drawn with TBtools (Chen et al., 2018).

To screen for the TFs potentially involved in the ethylene‐ or jasmonate‐regulated flavonoid biosynthesis pathway, we analysed the structures of the transcripts in the candidate DET set and detected 211 TFs (192 TFs at the gene level) from 41 TF families (Figure 8a), including genes encoding 21 ERF TFs, 20 NAC TFs, 20 MYB TFs (e.g. PpMYB10 and PpMYB114), 17 bHLH TFs, 11 WRKY TFs, 6 MYB‐related TFs, 6 b‐ZIP TFs, 6 CO‐like TFs, 3 EIL TFs, 3 MIKC_MADS TFs, 3 TCP TFs and 3 SBP TFs (Figure 8a). We subsequently analysed the TF gene expression levels following various treatments and clustered the genes into the following two groups (Figure 8b): group I, which contained most of the TFs in sets 1 and 3 and was positively responsive to MeJA + 1‐MCP and 1‐MCP treatments; group II, which contained most of the TFs in sets 2 and 4 and was positively responsive to ETH and MeJA treatments. These results imply that ethylene and jasmonate might regulate complex pear fruit coloration processes via a series of TFs comprising some of these candidate TFs.

## Discussion

### Single‐molecule long‐read sequencing unveils the complexity of the pear transcriptome

High‐throughput sequencing of cDNA is essential for genome annotations and gene discovery, but short‐read sequencing techniques rarely cover full transcripts. In contrast, FL cDNA sequencing enables researchers to obtain full‐length transcript sequences and reveal more alternatively spliced forms. Recently, FL cDNA sequencing has been applied to unveil the splice variants in diverse species. In maize, Wang *et al. *(2016) obtained 57% novel transcripts and identified many novel long noncoding RNAs (lncRNAs) via FL sequencing. The ‘Hongzaosu’ pear cultivar analysed in the current study is a hybrid of *P. pyrifolia* and *P. communis*, but the reported pear genome is that of *P. bretschneideri* (Wu *et al.*, 2013). An objective of our study was to maximize the transcript diversity of ‘Hongzaosu’ pear fruit skin under various conditions (light and hormones) by completing a PacBio sequencing experiment. Our analysis uncovered 20 458 novel isoforms of known genes and 1648 isoforms of novel genes (Figure S11A). Additionally, 309 lncRNAs and 235 fusion transcripts were identified (Figure 3f,g). These results verified the complexity of the ‘Hongzaosu’ pear genome and revealed considerable differences compared with the reported pear genome. By combining the novel transcripts and the reported pear genome, we enhanced the existing pear gene models and the pear reference sequence database to further elucidate the pear transcriptome.

### Ethylene inhibits anthocyanin biosynthesis by down‐regulating the expression of R2R3‐MYB genes and LBGs in red pears with diverse genetic backgrounds

Ethylene has long been considered an activator of anthocyanin biosynthesis in fruit tree crops because its production is highly related with fruit ripening, which is usually accompanied by anthocyanin accumulation. Specifically, ethylene‐induced red coloration has been observed in many rosaceous fruits, including apple (An *et al.*, 2017; Faragher and Brohier, 1984) and plum (Cheng *et al.*, 2016), as well as in the apple cultivar described herein (Figure S7). However, ethylene inhibited anthocyanin biosynthesis in red pear fruits with diverse genetic backgrounds (Figure 1 and Figures S4, S5 and S6). The inhibitory effect of ethylene on anthocyanin accumulation was previously observed in nonrosaceous species, including *A. thaliana*, cabbage and tobacco (Jeong *et al.*, 2010; Kang and Burg, 1973; Takada *et al.*, 2005). However, in this study, ethylene had the opposite effect on the analysed pear fruits, implying that the fruit coloration process differs between apple and pear fruits. Subsequent analyses revealed that ethylene inhibits anthocyanin biosynthesis by suppressing the expression of regulatory genes (*PpMYB10* and *PpMYB114*) and the LBGs (Figure 7).

An *et al.*(2017) described a regulatory mechanism underlying ethylene‐induced anthocyanin accumulation in apple fruit. The ethylene‐responsive TF MdEIL1 directly associates with *MdMYB1 *to up‐regulate expression, which increases anthocyanin biosynthesis. Although pear is genetically similar to apple, there are considerable differences between these two species. For example, because of the narrow genetic background of cultivated apple, the coloration of most red apple cultivars is due to the high expression of *MdMYB1/A/10*, which is located on chromosome 9. However, the genes responsible for the red coloration vary among pear cultivars. The *PpMYB114* gene (chromosome 5), whose apple ortholog has not been identified, is the only MYB gene that was identified from genetic analysis of red pears (Yao *et al.*, 2017). Another study confirmed that *PpMYB10* contributes to the coloration of red pears (Feng *et al.*, 2010). Accordingly, it is clear that the genetic regulation of the coloration of red pears is more complex than that of apple, which may be the main reason for the differences in the coloration patterns between these two species. Furthermore, the upstream regulators of these MYB genes are not well characterized. These MYB genes may be differentially regulated between the two species. Considering these two species responded differently to ethylene, it is reasonable to speculate that the diversity in the ethylene transduction pathway between the two species may be responsible for the differences in the red coloration patterns. In our study, we observed that *PpMYB10* and *PpMYB114* expression levels were down‐regulated by ethylene (Figure 7), indicating that ethylene inhibits anthocyanin biosynthesis through a *PpMYB10*‐ and *PpMYB114‐*related pathway. The EIN3/EIL1 proteins reportedly induce ethylene responses in apple and *A. Thaliana* (An *et al.*, 2017; Guo and Ecker, 2004). Considering that EIN3/EIL1 proteins are involved in all ethylene responses (Guo and Ecker, 2004), we speculated that EIN3/EIL1 proteins indirectly inhibit *PpMYB10* and *PpMYB114 *expression through other TFs, such as ERF and MYB TFs. The ERFs, which contain the EAR‐motif, are transcriptional repressors (Ohta *et al.*, 2001). Additionally, MYB repressors also regulate anthocyanin biosynthesis (Jeong *et al.*, 2010; Zhou *et al.*, 2019). Our analysis of the *MdMYB1*, *PpMYB10* and *PpMYB114* promoters revealed the presence of ERF‐binding sites only in the *PpMYB114* promoter. Thus, ethylene may inhibit pear anthocyanin biosynthesis through some ERF repressors that bind directly to the *PpMYB114* promoter. Furthermore, EIN3/EIL1 may post‐translationally regulate other TFs, such as MYB TFs as well as MYC and PIF TFs (both of which are bHLH TFs), and further suppress *PpMYB10* and *PpMYB114* transcription. Our RNA‐seq data revealed many ethylene‐responsive TFs (Figure 8) potentially related to anthocyanin biosynthesis. The possible functions of these TFs related to anthocyanin accumulation will be evaluated in detail in future studies.

### Jasmonate induces anthocyanin and flavone/isoflavone biosyntheses in red pear fruits

Jasmonate induces flavonoid accumulation in various plant species, such as *A. thaliana* and apple (An *et al.*, 2015; Qi *et al.*, 2011; Shan *et al.*, 2009). Our data indicated that jasmonate induces red coloration and anthocyanin accumulation in pear fruits. However, MeJA also activates ethylene production, which represses anthocyanin biosynthesis. Consequently, the inductive effect of jasmonate was observed only in the absence of ethylene (Figure 1 and Figure S4). The mechanism regulating jasmonate‐induced ethylene biosynthesis has been well characterized in apple. Specifically, jasmonate induces ethylene production by increasing MdMYC2 production, which activates the expression of *MdERF* and ethylene biosynthetic genes (Li *et al.*, 2017). These observations suggest that the relationship between jasmonate and ethylene production is conserved in apple and pear. Because ethylene and jasmonate enhance anthocyanin accumulation in apple, the MeJA + 1‐MCP treatment weakened the red pigmentation more than the MeJA treatment (Figure S7). In contrast, although MeJA did not activate the red pigmentation in pear fruits, the 1‐MCP treatment strongly enhanced anthocyanin biosynthesis, indicating that the ethylene‐mediated suppression of anthocyanin biosynthesis is greater than the inductive effect of jasmonate (Figure 1 and Figure S4). Additional analyses proved that jasmonate induces anthocyanin biosynthesis by up‐regulating LBG expression (Figure 7).

We also determined that jasmonate induces flavone/isoflavone biosynthesis in pear fruits (Figures 1 and 2 and Figure S4). Additionally, MeJA and MeJA + ETH treatments induce the deep yellow coloration of ‘Hongzaosu’ and ‘Mantianhong’ pear fruits, but not apple fruits (Figure 1 and Figures S4 and S5). Carotenoids and flavonoids are important pigments that produce a range of colours from yellow to red and from pale‐yellow to blue, respectively (Tanaka *et al.*, 2008). In plants, carotenoids and flavonoids are often present in the same organs, and their combined effects increase colour diversity (Tanaka *et al.*, 2008). Analyses of carotenoids and flavonoid metabolite profiles confirmed that the observed deep yellow coloration was due to flavone and isoflavone accumulation (Figures 1 and 2 and Figures S4 and S8).

In our study, MeJA induced the expression of *PpMYB10* and *PpMYB114* in the absence of ethylene (Figure 7). The anthocyanin biosynthesis structural genes had similar expression patterns, suggesting that the anthocyanin biosynthesis pathway is subjected to transcriptional regulation by a jasmonate‐responsive pathway. Furthermore, the expression levels of one *PpPAL*, one *PpCHS* and the cytochrome P450 (*PpFNS*/*PpIFS*) genes are positively responsive to ethylene and jasmonate and are positively correlated with yellow coloration (Figure 7). Therefore, jasmonate induces flavone/isoflavone production by up‐regulating the expression of flavone/isoflavone biosynthetic genes. Previous reports indicated that jasmonate activates flavonoid accumulation through the transcriptional and post‐translational regulation of MYB TFs. Jasmonate induces the degradation of MdJAZ proteins and releases MdbHLH3, which forms the MBW complex along with MdMYB9 and MdMYB11, while also directly up‐regulating *MdMYB9* and *MdMYB11* expression, ultimately leading to anthocyanin and PA biosyntheses (An *et al.*, 2015). On the basis of Mfuzz and co‐expression analyses, we selected 20 MYB TFs, of which 13 were positively responsive to jasmonate and negatively responsive to ethylene and seven were positively responsive to both jasmonate and ethylene (Figure 8). We also selected 17 bHLH TFs, of which nine were positively responsive to jasmonate and negatively responsive to ethylene and eight were positively responsive to both jasmonate and ethylene (Figure 8). These MYB and bHLH TFs may influence jasmonate‐induced anthocyanin or flavone/isoflavone biosynthesis, but the potential functions will need to be experimentally verified.

### Ethylene determines the branching of the jasmonate‐induced flavonoid pathway

Flavone/isoflavone and anthocyanin share common precursors. Accordingly, the flavone/isoflavone and anthocyanin levels are generally negatively correlated (Martens and Mithöfer, 2005). Specifically, decreases in anthocyanin levels will likely increase flavone/isoflavone accumulation because the precursors flow in only one direction. *Gerbera hybrida* ‘Th 58’, which has a heterozygous *Fns* locus (*fns* ^+^, *fns*), accumulates only 4′‐hydroxylated flavonoids, thus leading to orange coloration. In an earlier investigation, its self‐crossing population segregated into three groups with diverse FNS enzyme activities (high, medium and no activity). These groups exhibited yellow, orange and red coloration with low, medium and high anthocyanin contents, respectively (Martens and Forkmann, 2001; Martens and Mithöfer, 2005). In our study, jasmonate induced anthocyanin and flavone/isoflavone biosyntheses in red pear fruits (Figure 2c). However, ethylene determines the branching of the jasmonate‐induced flavonoid pathway. We observed that ethylene down‐regulated *PpF3′H* and *PpF3′5′H* expression levels (Figure 7), leading to the hyper‐accumulation of flavanone (Figure 2c and Table S2), which is the direct precursor of anthocyanin and flavone/isoflavone. Ethylene also blocked the expression of key anthocyanin biosynthetic genes (*PpANS* and *PpUFGT* genes), whereas jasmonate induced the expression of important flavone/isoflavone biosynthetic genes (*PpFNS* and *PpIFS*) (Figure 7). Finally, the pear skin accumulated a large amount of flavone/isoflavone and relatively little anthocyanin (Figures 1, 2 and Figure S4, Table S2). In contrast, when ethylene production was inhibited, the LBG expression levels were highly up‐regulated, which considerably induced the anthocyanin biosynthesis pathway (Figure 7). Finally, the pear skin accumulated a large amount of anthocyanin and relatively little flavone/isoflavone (Figures 1 and S4, Table S2). In *A. thaliana*, Zhu *et al.*(2011) proved that jasmonate can enhance the transcriptional activity of EIN3/EIL1 by removing JAZ proteins, which physically interact with and suppress EIN3/EIL1, and induce ethylene signalling. In the current study, *EIL* expression was up‐regulated by ETH and MeJA treatments (Figure 5 and Figure 8b), suggesting that jasmonate enhances ethylene signal transduction in pear, similar to its effects in *A. thaliana*.

**Figure 9:**
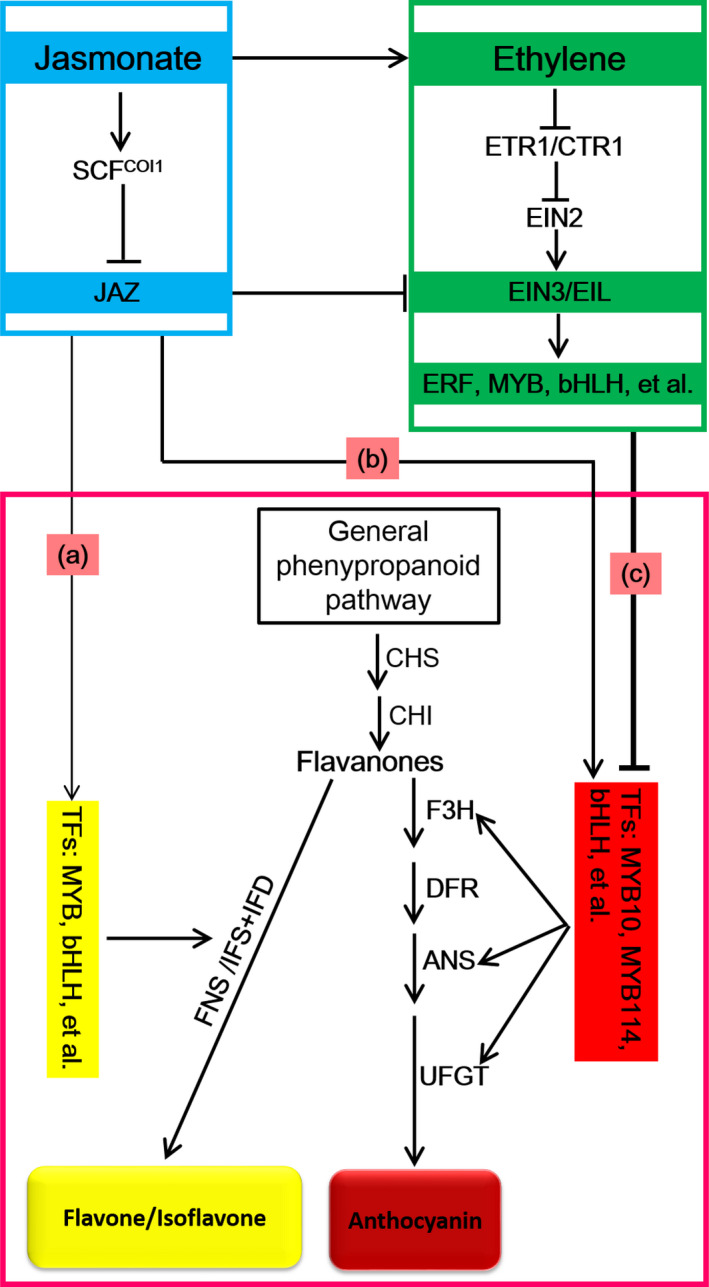
Putative model for ethylene‐inhibited anthocyanin biosynthesis and the decisive role of ethylene on the branching of the jasmonate‐induced flavonoid biosynthesis pathway in red Chinese pear. In the absence of ethylene, jasmonate induces the degradation of JAZ proteins and releases TFs (e.g. MYB and bHLH) that regulate flavone/isoflavone (a) or anthocyanin (b) biosynthesis. Flavone/isoflavone (a) and anthocyanin (b) biosynthesis pathways compete for the same precursors (flavanones) to determine fruit coloration (red or yellow). (c) Jasmonate induces ethylene production and ethylene signal transduction through EIN3/EIL1. Ethylene directly suppresses *PpMYB10* and *PpMYB114* expression via TF repressors (e.g. ERF, MYB and bHLH), ultimately inhibiting anthocyanin biosynthesis. This process leads to the accumulation of flavanones and provides abundant precursors for flavone/isoflavone biosynthesis. Thus, in the presence of ethylene, jasmonate induces flavone/isoflavone biosynthesis and the deep yellow coloration of pear fruits. The thickness of the line indicates the strength of the effect.

In summary, we determined that ethylene inhibits anthocyanin biosynthesis in red Chinese pear fruits, whereas jasmonate induces anthocyanin and flavone/isoflavone biosyntheses. Moreover, the branches of the jasmonate‐induced flavonoid pathway are determined by ethylene. We developed a potential underlying model based on our transcriptome analysis to elucidate the ethylene‐ and jasmonate‐regulated red pear coloration (Figure 9). In the absence of ethylene, jasmonate induces the degradation of JAZ proteins and releases TFs (e.g. MYB and bHLH), which regulate anthocyanin or flavone/isoflavone biosynthesis. Anthocyanin and flavone/isoflavone biosynthesis pathways compete for the same precursors (flavanones) to determine fruit coloration (i.e. red or yellow) (Figure 9a,b). However, jasmonate can induce ethylene production as well as ethylene signal transduction through EIN3/EIL1. Ethylene suppresses the expression of *PpMYB10* and *PpMYB114* via TF repressors (e.g. ERF, MYB and bHLH TFs) and ultimately inhibits anthocyanin biosynthesis (Figure 9c). This process leads to flavanone accumulation (Figure 2c) and provides abundant precursors for flavone/isoflavone biosynthesis. Thus, in the presence of ethylene, jasmonate induces flavone/isoflavone biosynthesis and the deep yellow coloration of pear fruits (Figure 9). Our data provide new insights into fruit colour phenotypes and the underlying regulatory mechanism controlled by jasmonate and ethylene. We also provide a transcriptome‐level perspective of the jasmonate‐ and ethylene‐regulated flavonoid biosynthesis pathway. The results of this study imply that the hormone‐regulated fruit coloration process is more complex than previously reported.

## Experimental procedures

### Plant materials and treatment

Fruits were collected from ‘Hongzaosu' (also called ‘Red Zaosu') pear (*Pyrus pyrifolia* × *Pyrus communis*) trees in the orchard of the Institute of Horticulture, Henan Academy of Agricultural Sciences, Henan, China. Fruits of ‘Mantianhong’ pear (*P. pyrifolia*) were sampled in a commercial orchard in Xiangyun, Yunnan, China. Fruits of ‘Nanhong’ pear (*P. ussuriensis*) were sampled in a commercial orchard in Xiongyue, Liaoning, China. Fruits of ‘Red Clapp’s Favorite’ pear (*P. communis*) were sampled in the orchard of the Yantai Academy of Agricultural Sciences in Yantai, Shandong, China. ‘Fuji' apple fruits were also collected from trees in the orchard of Northwest A & F University in Yangling, Shaanxi, China. All fruits were covered with lightproof double‐layered paper bags during the growing season as described by Huang *et al.* (2009). Bagged fruits were harvested at maturity [155 days after full bloom (DAFB)] and immediately transported to our laboratory.

Fruits were treated as indicated in Table 4. Specifically, the 1‐methylcyclopropene (1‐MCP; SmartFresh™) treatment was completed in sealed buckets at 25 °C for 16 h. The methyl jasmonate (MeJA) solution was dissolved in 1% ethanol. These solutions contained 0.01% Tween‐20 to promote absorption. Treated fruits were incubated for 10 days under continuous white light (60 μmol m^−2^ s^−1^) provided by overhead LEDs in a growth chamber set at 17 °C and 80% relative humidity. After measuring essential parameters, the pear fruit peel on the exposed side was collected, immediately frozen in liquid nitrogen, and stored at –80 °C until analysed.

**Table 4:** Groups of different treatments

Treatment	Concentration	Time	Replicates
dH_2_O (Control)	——	5 min	3
1% Ethanol (Control)	1%	5 min	3
1‐MCP	0.5 μl/l	16 h	3
MeJA (in 1% Ethanol) +1‐MCP	2 mM + 0.5 μl/l	5 min + 16 h	3
MeJA (in 1% Ethanol)	2 mM	5 min	3
MeJA (in 1% Ethanol)+ETH	2 mM + 2 mM	5 min	3
ETH	2 mM	5 min	3

### Extraction and measurement of total anthocyanins

The total anthocyanin content was measured using a modified version of the method described by Ni *et al.* (2017). Briefly, 0.2 g fruit peel was added to a 1.5‐ml solution comprising methanol:acetic acid (99:1, v/v) and incubated overnight at 4 °C. The absorbance at 530, 620 and 650 nm was measured with a DU800 spectrophotometer (Beckman Coulter, Fullerton, CA). The relative anthocyanin content was calculated as [(OD_530_ − OD_650_)−0.2 × OD_650_ − OD_620_)].

### Flavonoid metabolite profiling

The relative quantities of flavonoid metabolites in pear fruit peel samples were analysed with a liquid chromatography–electrospray ionization‐tandem mass spectrometry (LC‐ESI‐MS/MS) system by MetWare (Wuhan, China) (refer to Methods S1 for details regarding the protocol).

### RNA extraction, cDNA synthesis and quantitative real‐time PCR

Total RNA was extracted with a modified CTAB method (Zhang *et al.*, 2012). First‐strand cDNA was synthesized and a quantitative real‐time (qRT)‐PCR assay was conducted as described by Ni *et al. *(2019), with primers designed with an online tool (https://www.ncbi.nlm.nih.gov/tools/primer-blast/) (Table S1).

### Illumina RNA‐seq library construction

Total RNA was extracted from control (1% ethanol) samples as well as samples treated with ethephon (ETH), 1‐MCP, MeJA and MeJA + 1‐MCP, with three biological replicates per sample. The RNA concentration was measured using a NanoDrop 2000 spectrophotometer (Thermo, Waltham, MA), whereas RNA integrity was assessed with the RNA Nano 6000 Assay Kit and the 2100 Bioanalyzer system (Agilent Technologies, Santa Clara, CA). For each sample, 1 μg RNA was used as the template for preparing RNA‐seq libraries with the NEBNext Ultra™ RNA Library Prep Kit for Illumina (NEB, Beverly, MA). Index codes were added to attribute sequences to each sample. Library quality was assessed with the 2100 Bioanalyzer system. The index‐coded samples were clustered with the cBot Cluster Generation System and the TruSeq PE Cluster Kit v4‐cBot‐HS (Illumina). The libraries were sequenced with the HiSeq Xten platform (Illumina, San Diego, CA) by Biomarker (Beijing, China).

### Library construction and Pacific Biosciences long‐read sequencing

The RNA extracted from each sample was combined, and 1 μg RNA was reverse transcribed with the SMARTer™ cDNA synthesis kit (Clontech, Mountain View, CA) to generate barcoded FL cDNA. Three barcoded SMRTBell libraries (1–2, 2–3 and 3–6 kb) were generated with the BluePippin system (Sage, Beverly, MA) to remove trace amounts of small inserts. The libraries were sequenced with the PacBio sequencing system, which generated 19.78 Gb of clean reads. Raw reads were processed into error‐corrected reads of inserts with the default parameters of the ToFu pipeline. The resulting FL nonchimeric transcripts were detected by searching for the poly‐A tail signal and the 5′ and 3′ cDNA primer target sites in reads of inserts. The iterative clustering for error correction was used to obtain consensus isoforms, and FL consensus sequences were polished with Quiver. Full‐length transcripts with a postcorrection accuracy above 99% were generated and analysed further (Figure S1).

### Pacific Biosciences long‐read sequencing data analysis

The FL consensus sequences were mapped to a reference genome (Wu *et al.*, 2013) with GMAP (Wu and Watanabe, 2005). Mapped reads were further collapsed with the pbtranscript‐ToFU package (min‐coverage = 85% and min‐identity = 90%). The 5′ differences were ignored when collapsing redundant transcripts (Figure S1). Transcripts were validated against reference transcript annotations with the Python script MatchAnnot. The AS events, including IR, ES, AD, AA and MEE, were identified with AStalavista. All transcripts were annotated based on the following public databases: Nr (NCBI nonredundant protein sequences), Pfam (protein families), KOG/COG/eggNOG (Clusters of Orthologous Groups of proteins), Swiss‐Prot (manually annotated and reviewed protein sequences), KEGG (Kyoto Encyclopedia of Genes and Genomes) and GO (Gene Ontology).

### RNA‐seq data analysis

Raw data (raw reads) in the fastq format were first processed with in‐house Perl scripts. Clean data (clean reads) were obtained from the raw data by removing reads containing an adapter or poly‐N sequences as well as low‐quality reads. Moreover, the Q20, Q30, GC‐content and sequence duplication level of the clean data were calculated. All subsequent analyses were completed with the high‐quality clean data. The clean reads were then mapped to the reference genome with TopHat2. Only reads with a perfect match or one mismatch were used for mapping.

Expression levels were estimated based on the number of fragments per kilobase of transcript per million reads mapped (FPKM). Differential expression between two conditions/groups was analysed with the DESeq R package (1.10.1). The resulting *P*‐values were adjusted according to the Benjamini and Hochberg approach for controlling the false discovery rate. Genes with an adjusted *P*‐value < 0.05 detected with DESeq were designated as differentially expressed genes (fold‐change ≥ 2, false discovery rate < 0.01).

### Statistical analysis

Data were subjected to a one‐way ANOVA, with treatment means separated by Tukey's multiple range test in the Statistical Product and Service Solutions program (version 19) (SPSS Inc., Chicago, IL). The LSDs (α = 0.05) were calculated with the Data Processing System (version 3.01) (Zhejiang University, Hangzhou, China).

### Accession codes

The sequencing data have been deposited in the NCBI Sequence Read Archive (http://www.ncbi.nlm.nih.gov/sra/), with the BioProject IDPRJNA544971.

## Author contributions

J.N., S.B. and Y.T. conceived and planned the study. J.N., L.G., J. Li., Y. Li and R.T. collected the samples. J.N. and L.Y. extracted the total RNA. J.N., Y.Z., J.S. and M.Q. profiled the flavonoid metabolites, analysed the pigment content and sugar/acid content. J.N. completed the qRT‐PCR assay and bioinformatics analysis. J.N., Å.S., S.B. and Y.T. wrote the manuscript. All authors read and approved the final manuscript.

## Conflict of interests

The authors declare that they have no competing interests.

## Supporting information


**Figure S1** Workflow for Pacific Biosciences (PacBio) Iso‐Seq data processing.
**Figure S2** Effects of various treatments on the (A) chroma, (B) lightness (L*), (C) hue angle (Hue) and (D) b* value of ‘Hongzaosu’ fruits following white light irradiation. Data are presented as the mean ± standard error of three biological replicates.
**Figure S3 **Effects of various treatments on the (A) fructose, (B) glucose, (C) sorbitol, (D) sucrose, (E) malic acid, and (F) citric acid contents of ‘Hongzaosu’ fruits following a white light irradiation. Data are presented as the mean ± standard error of three biological replicates.
**Figure S4** Effects of various treatments on the coloration of ‘Mantianhong’ Chinese sand pear fruits.
**Figure S5 **Effects of ethephon and 1‐MCP treatments on the coloration of ‘Nanhong’ pear fruits.
**Figure S6** Effects of ethephon and 1‐MCP treatments on the coloration of ‘Red Clapp's Favorite' pear fruits.
**Figure S7** Effects of various treatments on the coloration of ‘Fuji’ apple fruits.
**Figure S8** (A) Effects of different treatments on the total carotenoid contents of ‘Hongzaosu’ pear fruit peels. (B) Carotenoid compounds in the samples exposed to the control, 1‐MCP and MeJA treatments were analysed and quantified by LC‐MS/MS. Data are presented as the mean ± standard error of three biological replicates.
**Figure S9** Principle component analysis plots of the first and second components of diverse treatments of ‘Hongzaosu’ pear fruit peels.
**Figure S10** Quantification of three size‐fractionated libraries (A–C) and the size range of each library (D).
**Figure S11** (A) Comparison of PacBio and pear genome isoforms. (B) Comparison of the isoform length density between the PacBio and pear genome. (C) Various types of alternative splicing events in the ‘Hongzaosu’ pear fruit peels.
**Figure S12** Validation of the split gene model for PacBio Iso‐Seq.
**Figure S13** Verification of alternative splicing events in three genes by PCR.
**Figure S14** Comparison of the diﬀerentially expressed transcripts in each sample pair.
**Figure S15** Validation of differentially expressed transcripts by qRT‐PCR.
**Figure S16** Distribution of enriched KEGG pathways for various gene expression patterns in response to hormones.
**Figure S17** Hierarchical cluster tree with 28 modules of co‐expressed genes.

**Table S1** Details regarding the qRT‐PCR primers.
**Table S2** Enriched KEGG pathways and the relative abundance of differentially accumulated flavonoid metabolites.
**Table S3 **Details regarding the full‐length sequences.
**Table S4** Details regarding the RNA‐seq data.
**Table S5** Transcripts in different clusters following the Mfuzz analysis.
**Table S6 **Transcripts in different sets.

**Methods S1** Flavonoid metabolite profiling.
