# Supplementary material for: Ethylene mediates the branching of the jasmonate‐induced flavonoid biosynthesis pathway by suppressing anthocyanin biosynthesis in red Chinese pear fruits
Source: Plant Biotechnol J. 2019 Nov 19;18(5):1223–40. doi: 10.1111/pbi.13287 (PMC7152598; doi:10.1111/pbi.13287)
Supplement: Supplementary file 1 — Figure S1 Workflow for Pacific Biosciences (PacBio) Iso‐Seq data processing. Figure S2 Effects of various treatments on the (A) chroma, (B) lightness (L*), (C) hue angle (Hue) and (D) b* value of ‘Hongzaosu’ fruits following white light irradiation. Data are presented as the mean ± standard error of three biological replicates. Figure S3 Effects of various treatments on the (A) fructose, (B) glucose, (C) sorbitol, (D) sucrose, (E) malic acid, and (F) citric acid contents of ‘Hongzaosu’ fruits following a white light irradiation. Data are presented as the mean ± standard error of three biological replicates. Figure S4 Effects of various treatments on the coloration of ‘Mantianhong’ Chinese sand pear fruits. Figure S5 Effects of ethephon and 1‐MCP treatments on the coloration of ‘Nanhong’ pear fruits. Figure S6 Effects of ethephon and 1‐MCP treatments on the coloration of ‘Red Clapp's Favorite' pear fruits. Figure S7 Effects of various treatments on the coloration of ‘Fuji’ apple fruits. Figure S8 (A) Effects of different treatments on the total carotenoid contents of ‘Hongzaosu’ pear fruit peels. (B) Carotenoid compounds in the samples exposed to the control, 1‐MCP and MeJA treatments were analysed and quantified by LC‐MS/MS. Data are presented as the mean ± standard error of three biological replicates. Figure S9 Principle component analysis plots of the first and second components of diverse treatments of ‘Hongzaosu’ pear fruit peels. Figure S10 Quantification of three size‐fractionated libraries (A–C) and the size range of each library (D). Figure S11 (A) Comparison of PacBio and pear genome isoforms. (B) Comparison of the isoform length density between the PacBio and pear genome. (C) Various types of alternative splicing events in the ‘Hongzaosu’ pear fruit peels. Figure S12 Validation of the split gene model for PacBio Iso‐Seq. Figure S13 Verification of alternative splicing events in three genes by PCR. Figure S14 Comparison of the diﬀerentially expressed trans [file PBI-18-1223-s003.pdf]

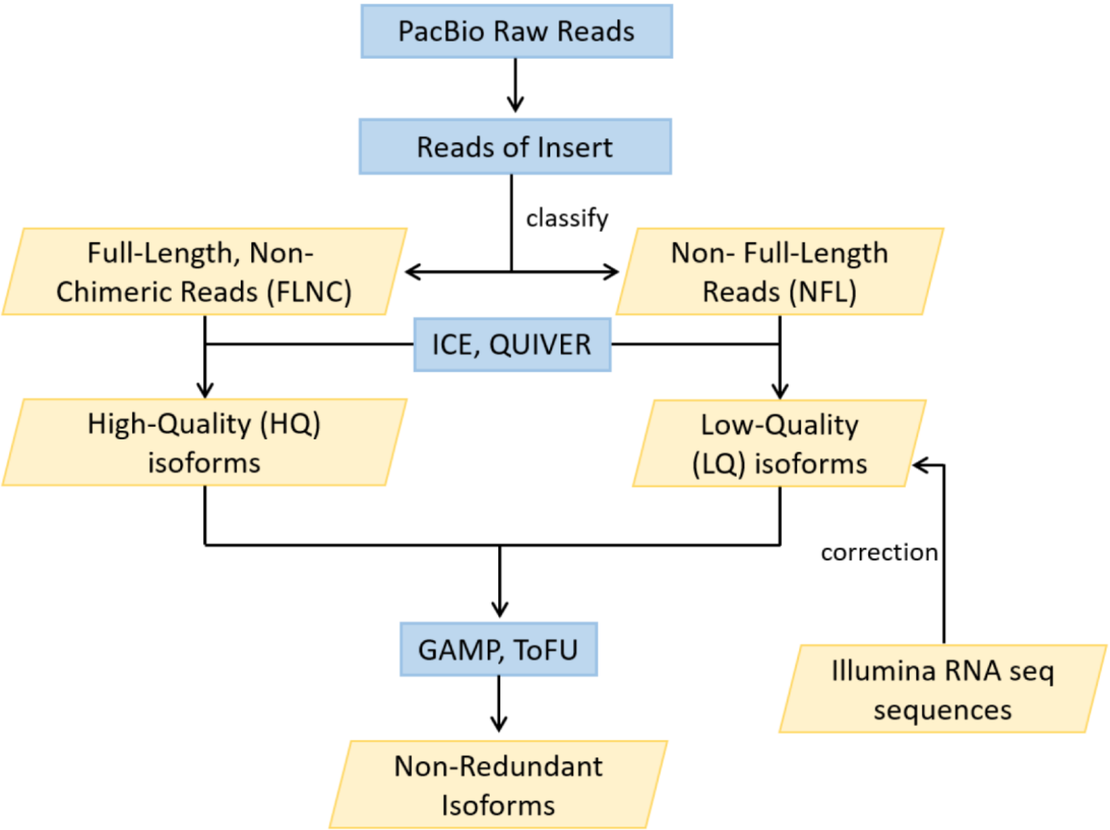

Fig. S1 Workflow for Pacific Biosciences (PacBio) Iso-Seq data processing.

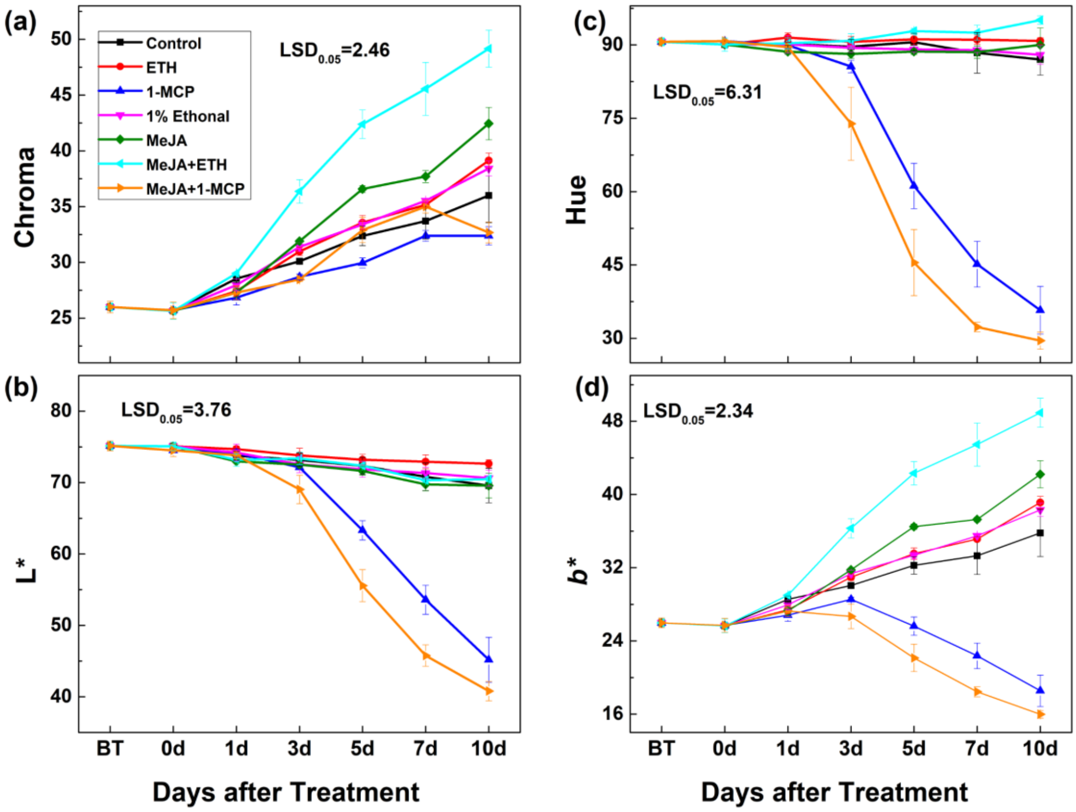

Fig. S2 Effects of various treatments on the (A) chroma, (B) lightness (*L**), (C) hue angle (*Hue*), and (D) *b** value of 'Hongzaosu' fruits following white light irradiation. Data are presented as the mean ± standard error of three biological replicates.

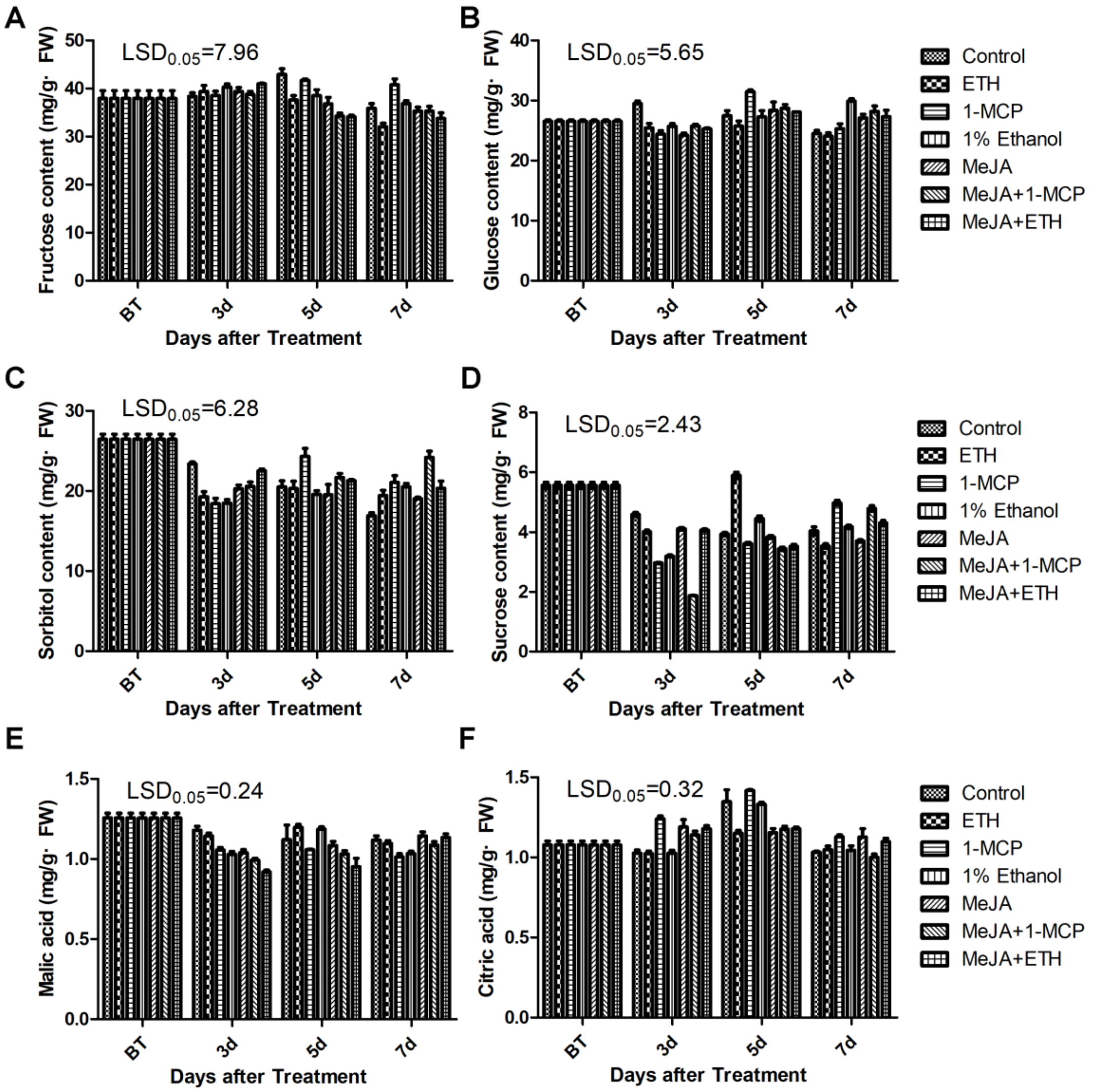

Fig. S3 Effects of various treatments on the (A) fructose, (B) glucose, (C) sorbitol, (D) sucrose, (E) malic acid, and (F) citric acid contents of 'Hongzaosu' fruits following a white light irradiation. Data are presented as the mean ± standard error of three biological replicates.

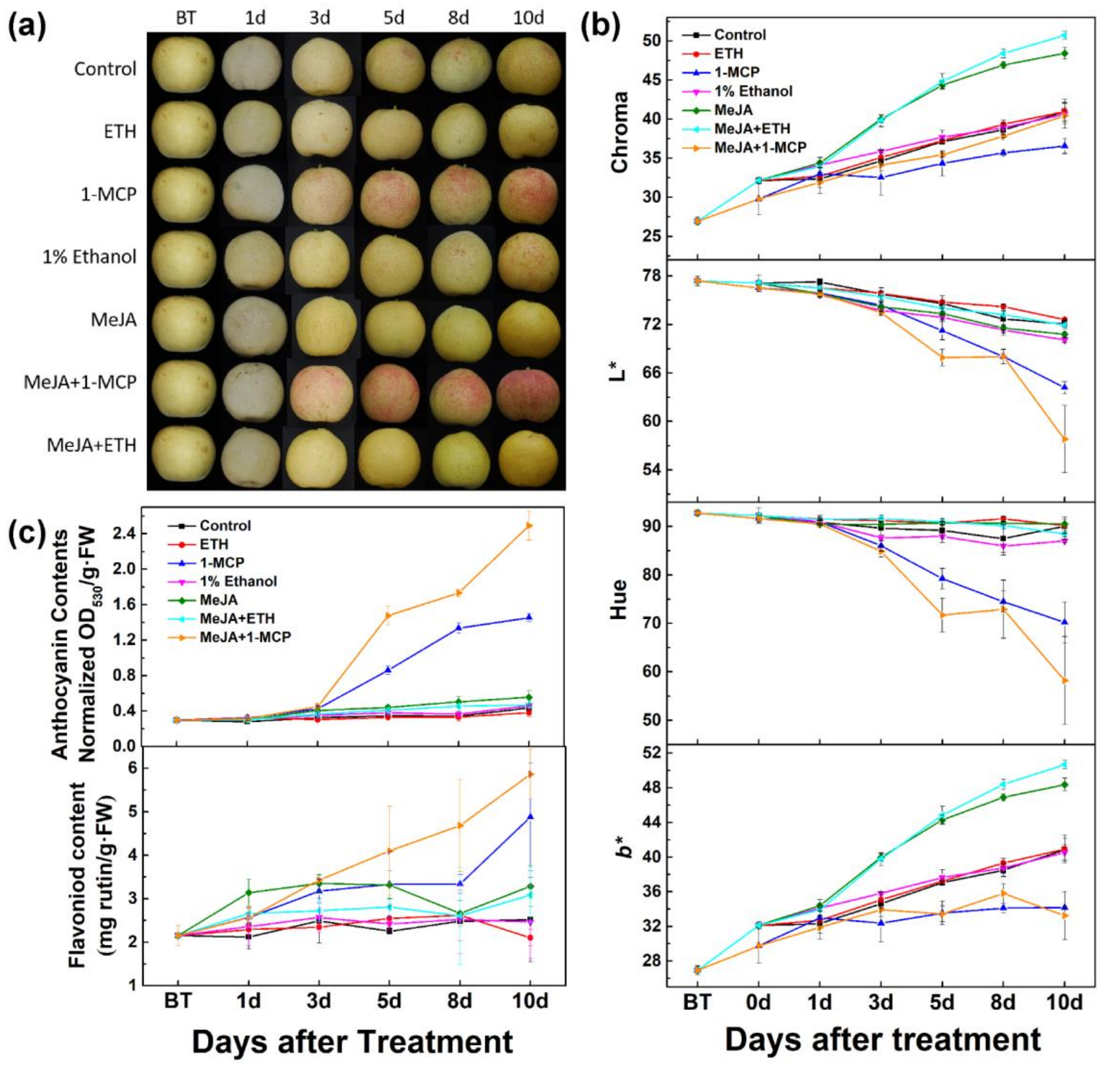

Fig. S4 Effects of various treatments on the coloration of 'Mantianhong' Chinese sand pear fruits. (A) Representative images of 'Mantianhong' pear fruits following diverse treatments. (B) Effects of various treatments on the chroma, lightness (*L**), hue angle (*Hue*), and *b** value of 'Mantianhong' fruits. (C) Effects of diverse treatments on the total anthocyanin content and total flavonoid content. ETH, fruit treated with ethephon; MeJA, fruit treated with methyl jasmonate; 1-MCP, fruit treated with 1-methylcyclopropene. Data are presented as the mean ± standard error of three biological replicates.

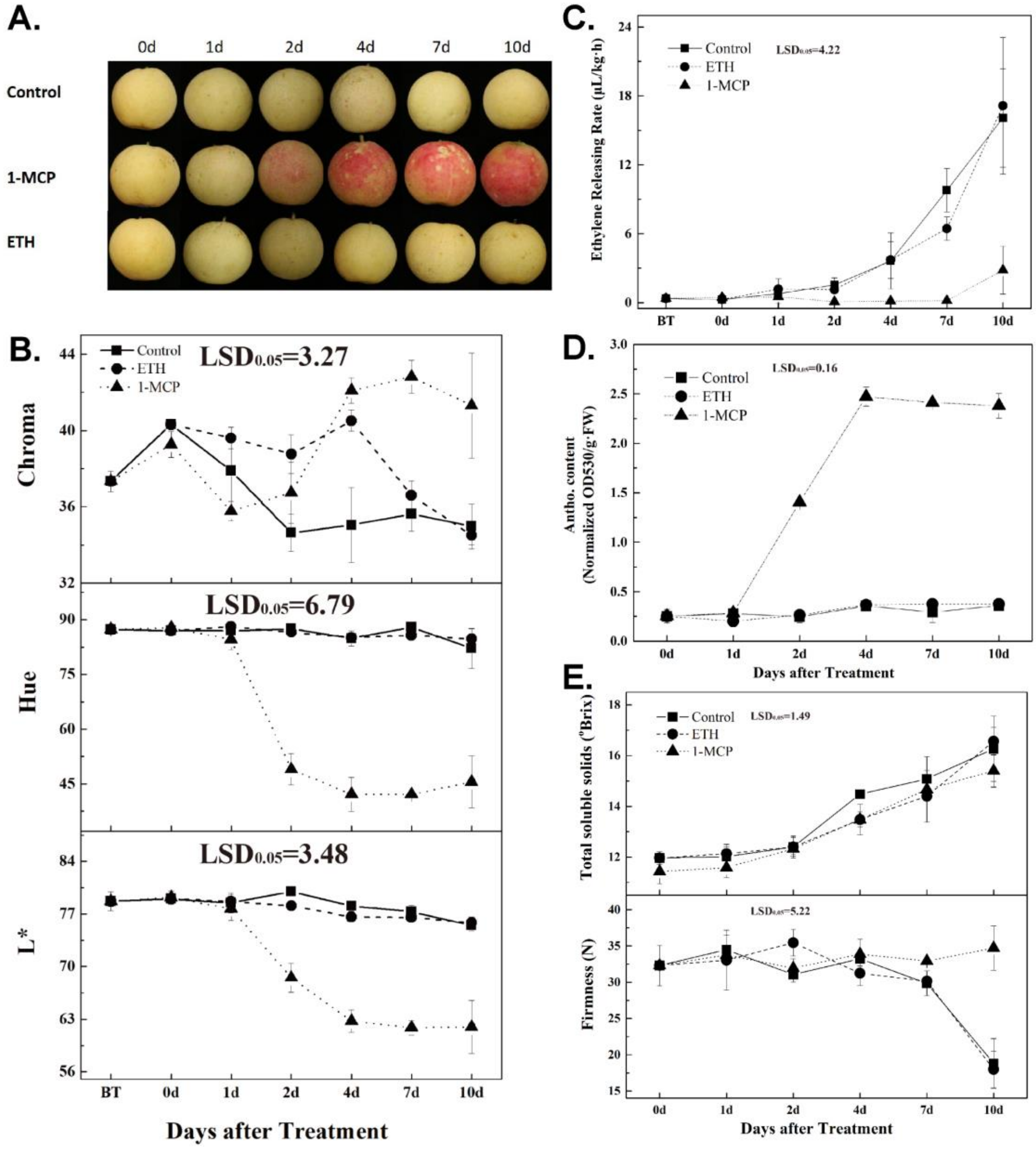

Fig. S5 Effects of ethephon and 1-MCP treatments on the coloration of 'Nanhong' pear fruits. (A) Representative images of 'Nanhong' pear fruits following diverse treatments. (B) Effects of various treatments on the chroma, lightness (*L**), hue angle (*Hue*), and *b** value of 'Nanhong' pear fruits. (C) Effects of diverse treatments on the ethylene-release rate. (D) Effects of diverse treatments on the total anthocyanin content. (E) Effects of diverse treatments on the TSS content and firmness of 'Nanhong' pear fruits. Data are presented as the mean ± standard error of three biological replicates.

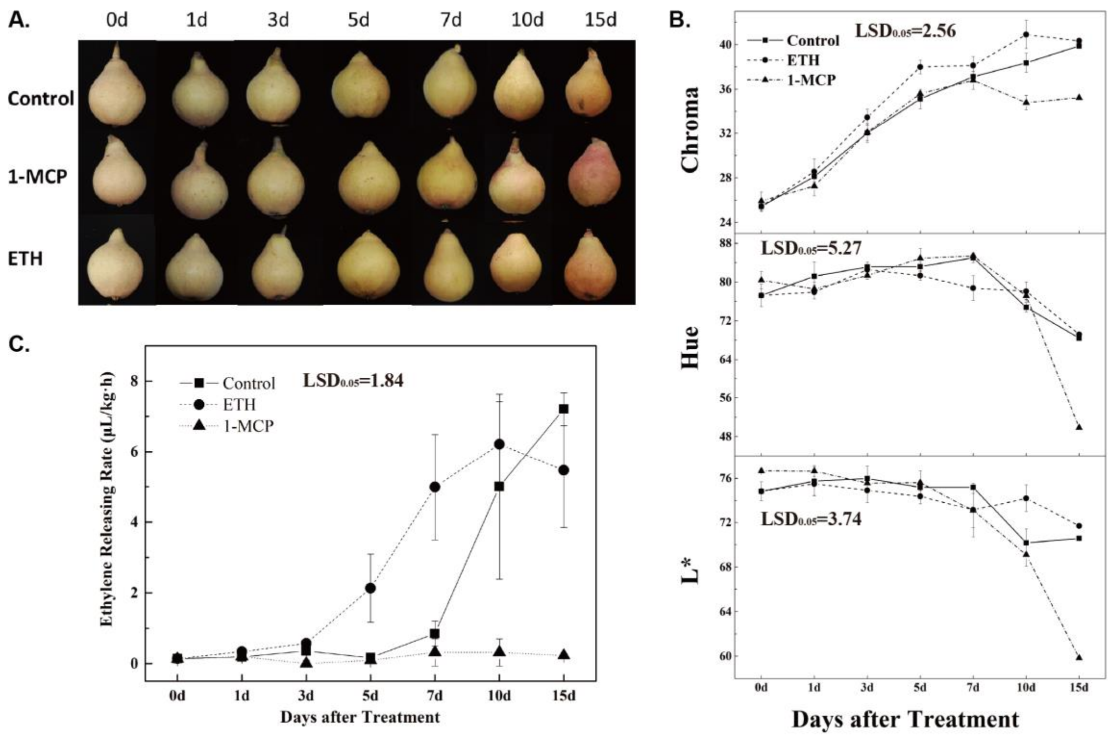

Fig. S6 Effects of ethephon and 1-MCP treatments on the coloration of 'Red Clapp's Favorite' pear fruits. (A) Representative images of 'Red Clapp's Favorite' pear fruits following diverse treatments. (B) Effects of various treatments on the chroma, lightness (L*), hue angle (*Hue*), and b* value of 'Red Clapp's Favorite' pear fruits. (C) Effects of diverse treatments on the ethylene-release rate. Data are presented as the mean ± standard error of three biological replicates.

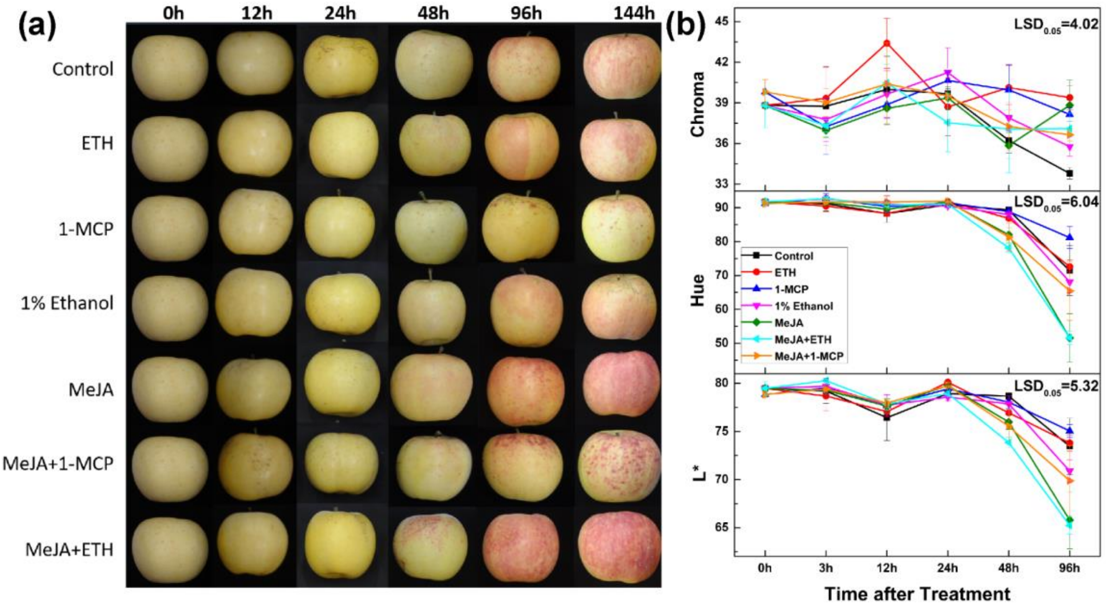

Fig. S7 Effects of various treatments on the coloration of 'Fuji' apple fruits. (A) Photos of 'Fuji' apple fruits following diverse treatments. (B) Effects of various treatments on the chroma, lightness (*L**), and hue angle (*Hue*) of 'Fuji' apple fruits. Data are presented as the mean ± standard error of three biological replicates.

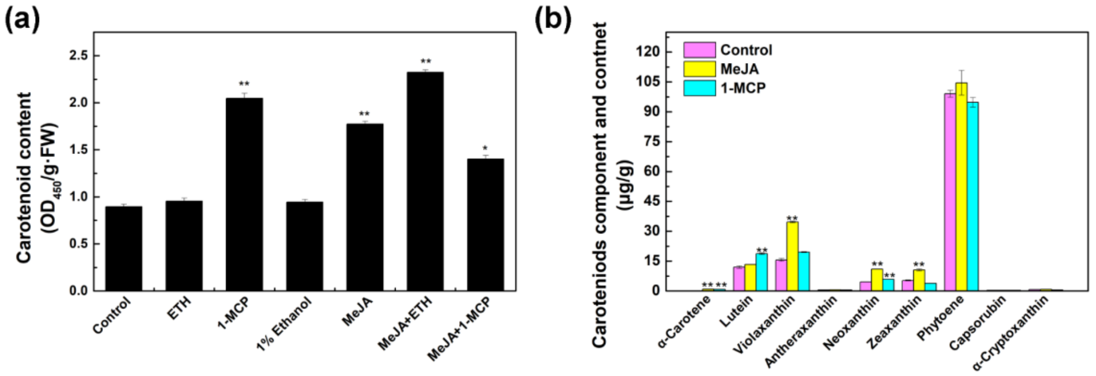

Fig. S8 (A) Effects of different treatments on the total carotenoid contents of 'Hongzaosu' pear fruit peels. (B) Carotenoid compounds in the samples exposed to the control, 1-MCP, and MeJA treatments were analyzed and quantified by LC-MS/MS. Data are presented as the mean ± standard error of three biological replicates.

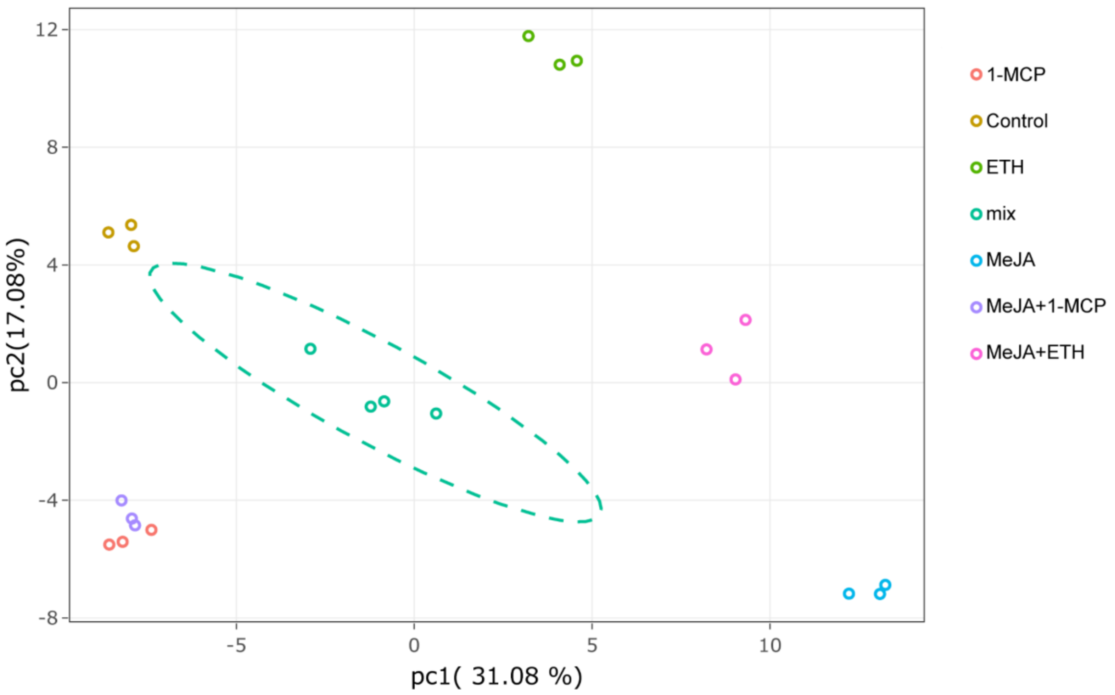

Fig. S9 Principle component analysis plots of the first and second components of diverse treatments of 'Hongzaosu' pear fruit peels.

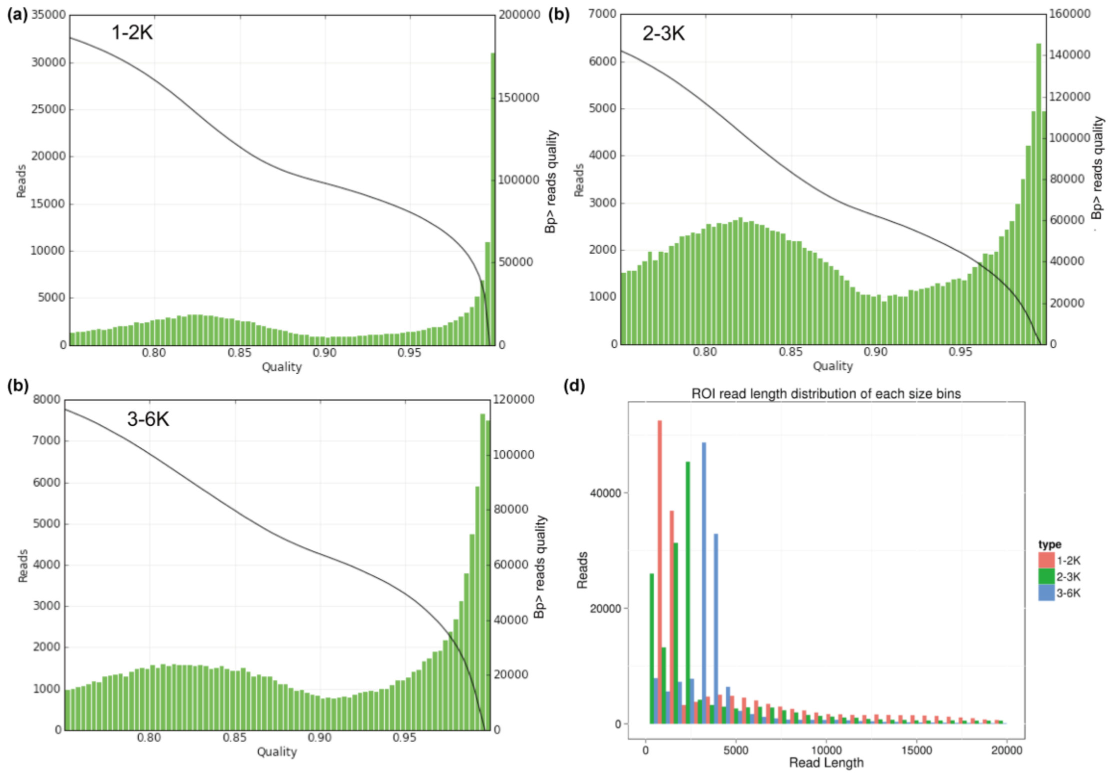

Fig. S10 Quantification of three size-fractionated libraries (A–C) and the size range of each library (D).

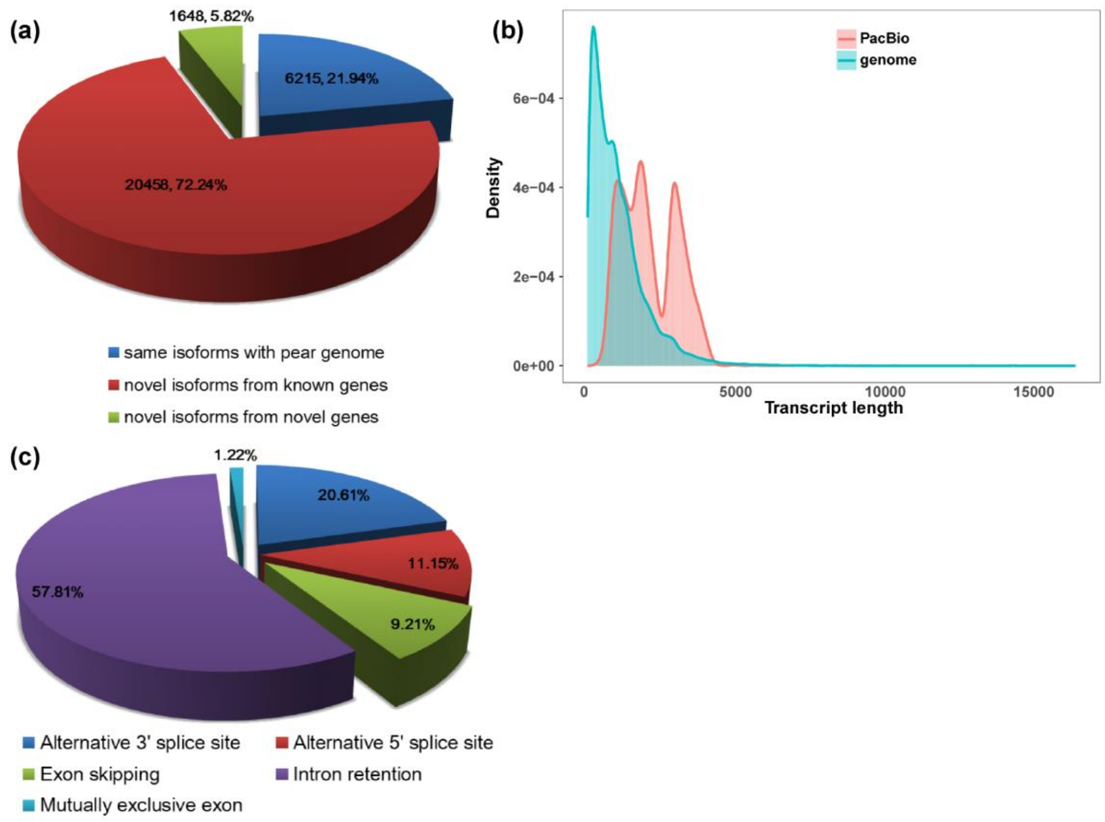

Fig. S11 (A) Comparison of Pacific Biosciences (PacBio) and pear genome isoforms. (B) Comparison of isoform length density between the PacBio and pear genome. (C) Various types of alternative splicing events in the 'Hongzaosu' pear fruit peels.

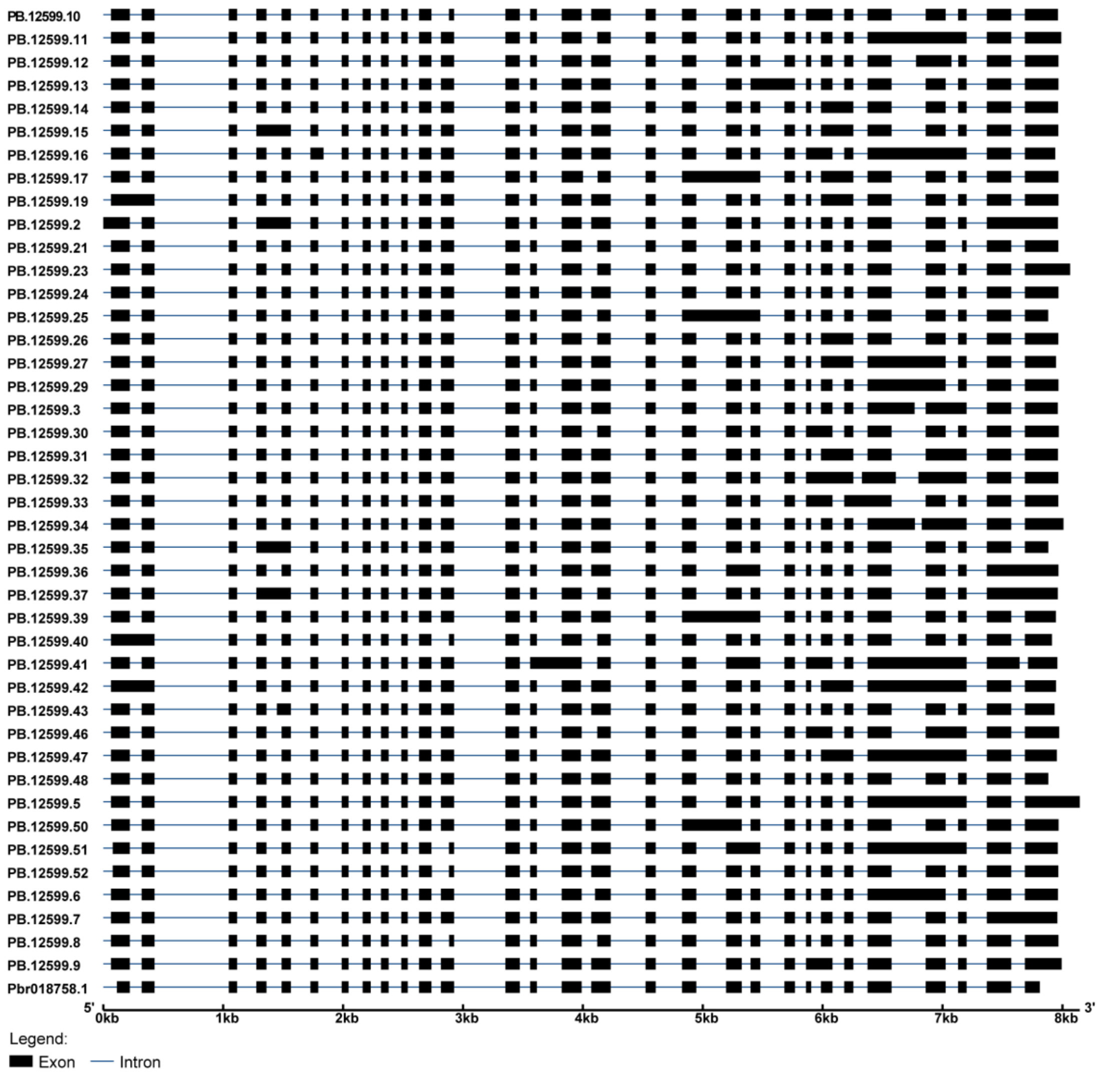

Fig. S12 Validation of the split gene model for Pacific Biosciences (PacBio) Iso-Seq.

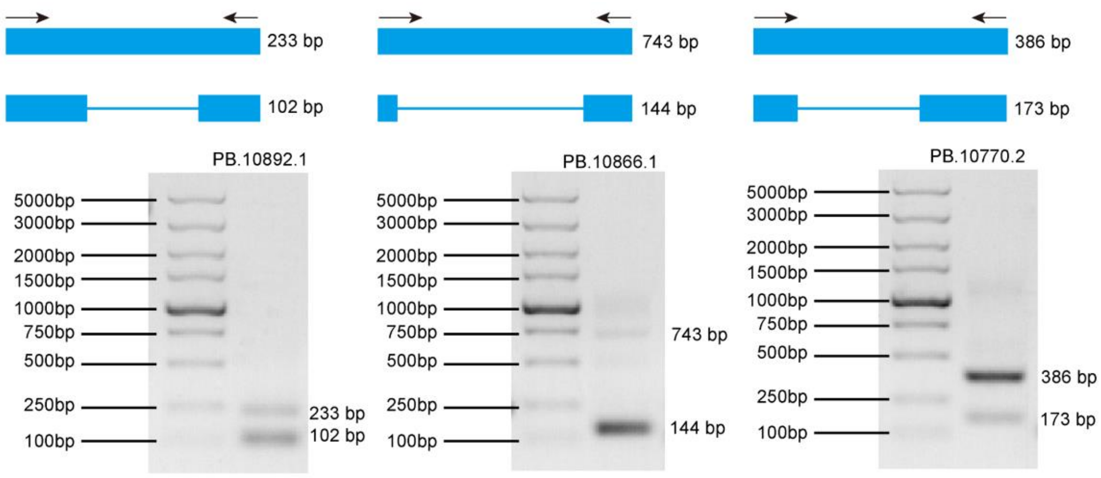

Fig. S13 Verification of alternative splicing events in three genes by PCR.

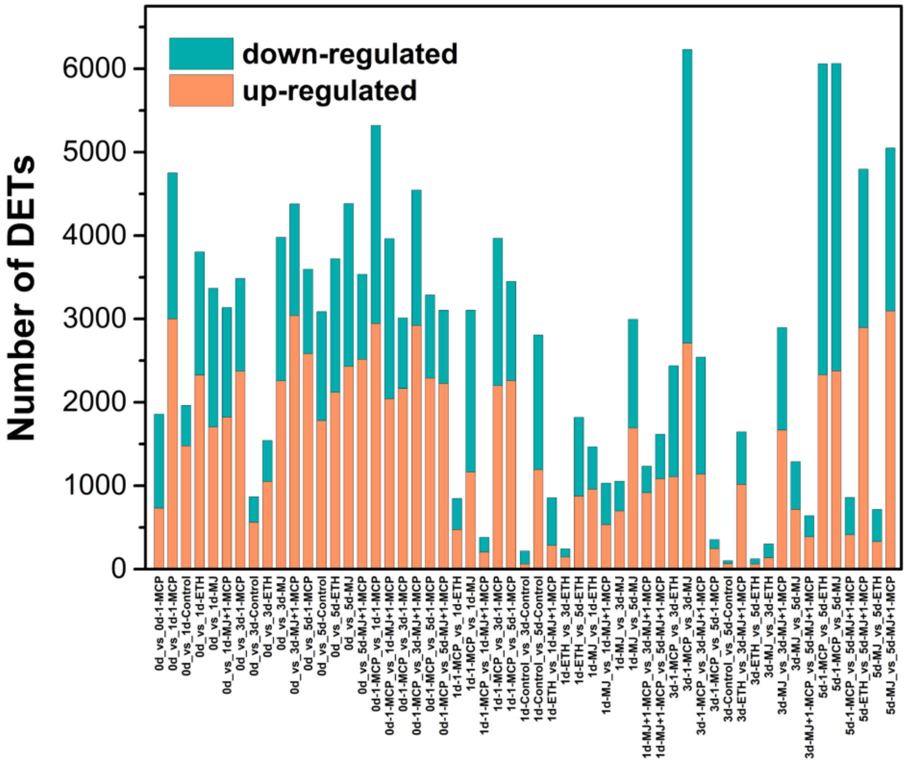

Fig. S14 Comparison of the differentially expressed transcripts in each sample pair.

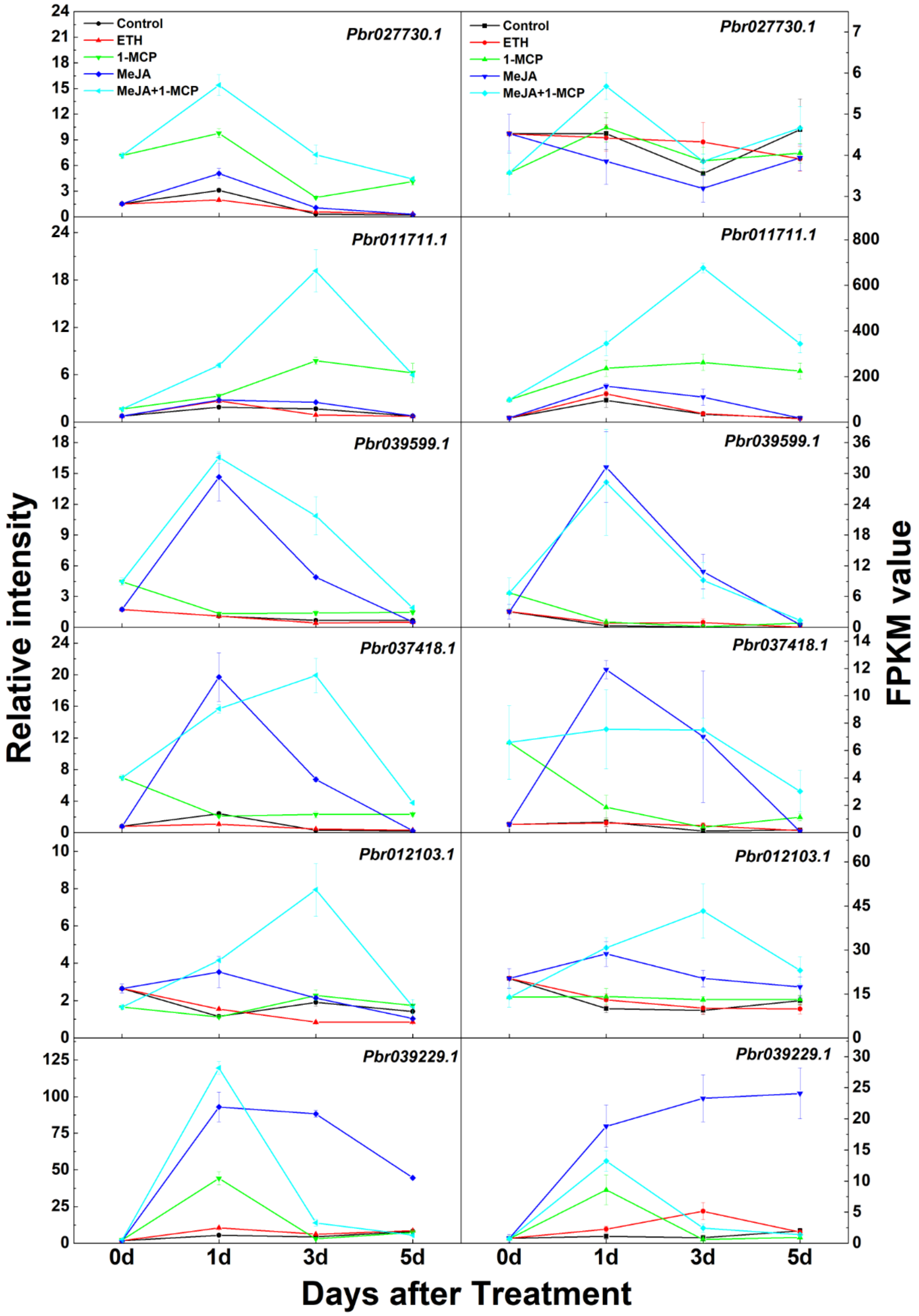

Fig. S15 Validation of differentially expressed transcripts by qRT-PCR. Data are presented as the mean ± standard error of three biological replicates.

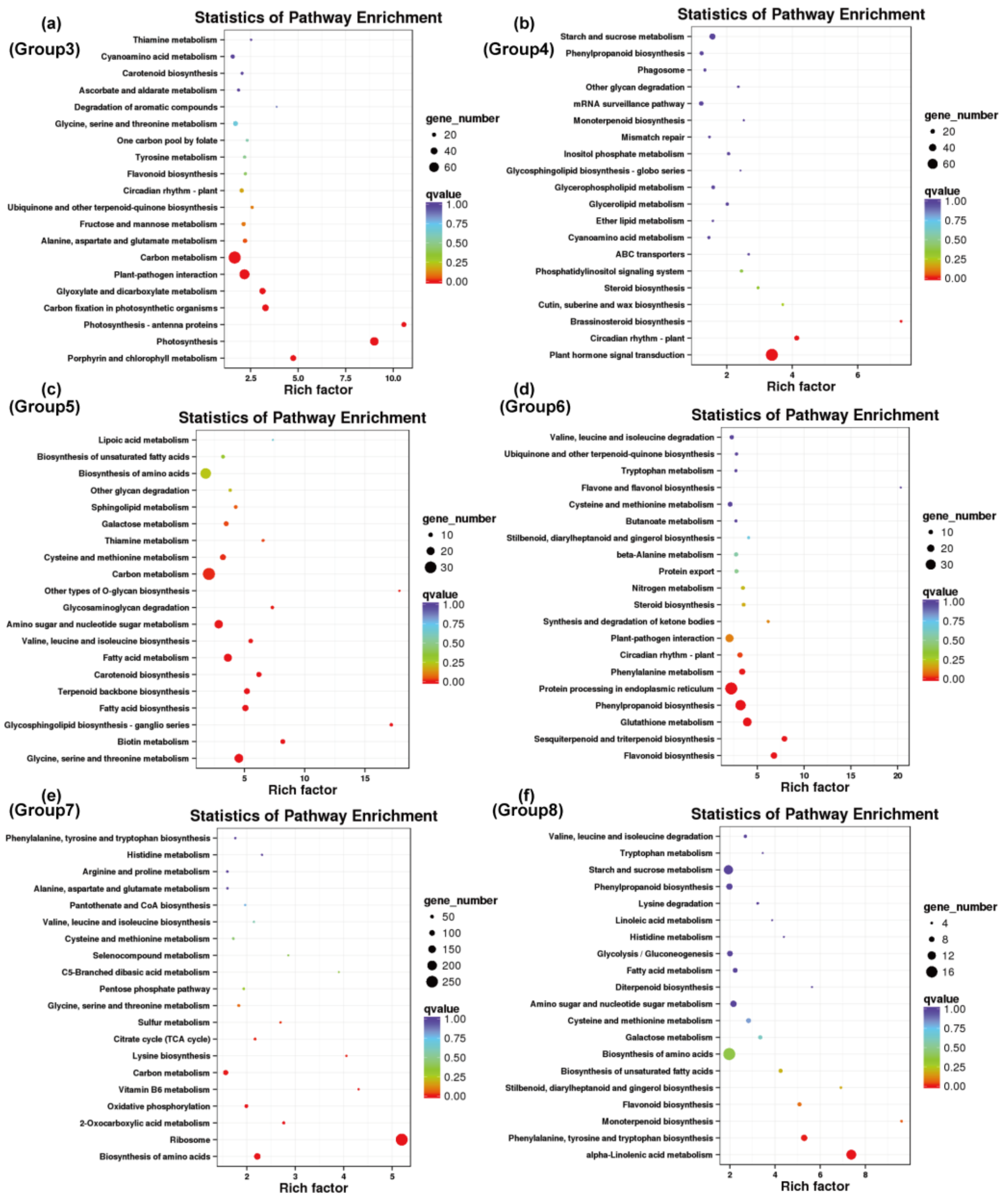

Fig. S16 Distribution of enriched KEGG pathways for various gene expression patterns in response to hormones. (A) Group 3, transcripts that negatively responded to ethylene, including clusters 5, 6, 11, and 20. (B) Group 4, transcripts that rapidly negatively responded to ethylene, including clusters 7, 15, and 18. (C) Group 5, transcripts that positively responded to ethylene, including cluster 17. (D) Group 6, transcripts that positively responded to jasmonate and negatively responded to ethylene, including clusters 10 and 19. (E) Group 7, transcripts that positively responded to ethylene (dominant) and jasmonate, including clusters 8, 13, and 16. (F) Group 8, transcripts that positively responded to ethylene and jasmonate (dominant), including cluster 12. Each circle represents a KEGG pathway.

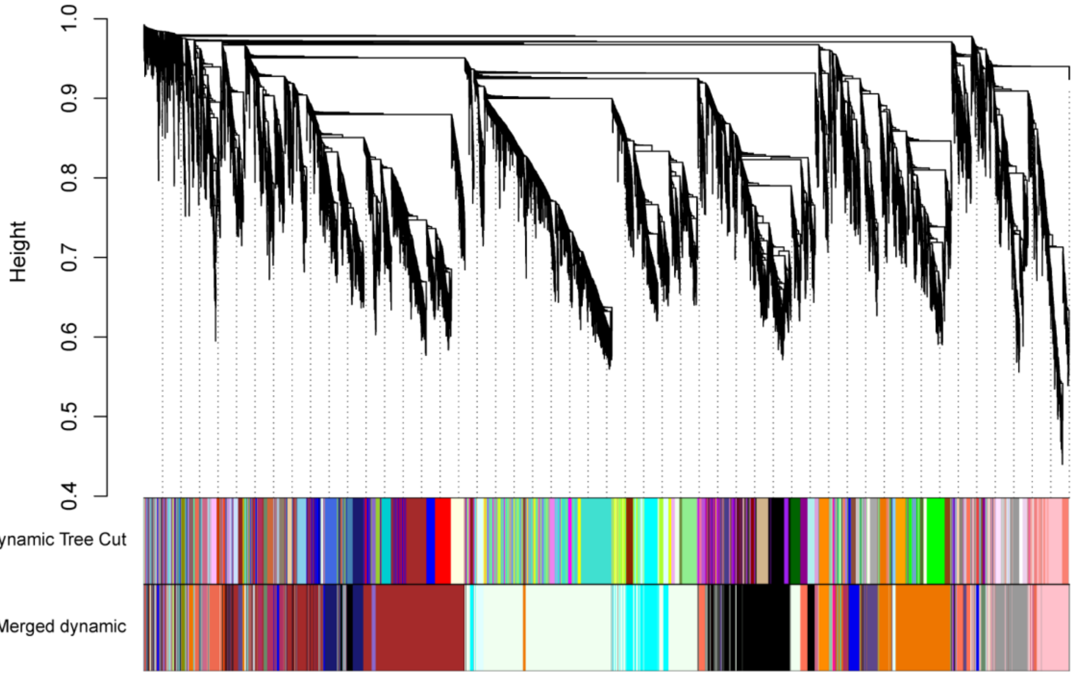

Fig. S17 Hierarchical cluster tree with 28 modules of co-expressed genes. The lower panel presents the modules in designated colors.
