# Supplementary material for: Ethylene mediates the branching of the jasmonate‐induced flavonoid biosynthesis pathway by suppressing anthocyanin biosynthesis in red Chinese pear fruits
Source: Plant Biotechnol J. 2019 Nov 19;18(5):1223–40. doi: 10.1111/pbi.13287 (PMC7152598; doi:10.1111/pbi.13287)
Supplement: Supplementary file 3 — Methods S1 Flavonoid metabolite profiling. [file PBI-18-1223-s002.docx]

**Flavonoid metabolite profiling**

A liquid chromatography–electrospray ionization-tandem mass spectrometry (LC-ESI-MS/MS) system was used for the relative quantification of flavonoid metabolites in pear fruit peel samples. The fruit peel samples (0.1 g powder) were treated with 1.0 mL 70% aqueous methanol overnight at 4 ℃. After a centrifugation at 10,000 × g for 10 min, the supernatants were collected (CNWBOND Carbon-GCB SPE Cartridge, 250 mg, 3 mL; ANPEL, Shanghai, China), filtered (SCAA-104, 0.22 μm pores; ANPEL), and analyzed with an LC-ESI-MS/MS system comprising the Shim-pack UFLC CBM30A system (Shimadzu) and the 6500 Q TRAP system (Applied Biosystems). The analytical conditions were as follows: LC: column, Waters ACQUITY UPLC HSS T3 C18 (1.8 µm, 2.1 mm × 100 mm); solvent system, water (0.04% acetic acid):acetonitrile (0.04% acetic acid); gradient program, 100:0 (v/v) at 0 min, 5:95 (v/v) at 11.0 min, 5:95 (v/v) at 12.0 min, 95:5 (v/v) at 12.1 min, and 95:5 (v/v) at 15.0 min; flow rate, 0.40 mL/min; temperature, 40 °C; injection volume: 2 μL. The effluent was analyzed with an ESI-triple quadrupole-linear ion trap (Q TRAP) system. The LIT and triple quadrupole (QQQ) scans were acquired on a 6500 Q TRAP LC-MS/MS system equipped with an ESI Turbo Ion-Spray interface. The system was operated in the positive ion mode and controlled with the Analyst 1.6.3 program (AB Sciex). Instrument tuning and mass calibration were completed with 10 and 100 μmol/L polypropylene glycol solutions in QQQ and LIT modes, respectively. The QQQ scans were acquired as MRM experiments with the collision gas (nitrogen) set to 5 psi. The DP and CE were optimized for individual MRM transitions. A specific set of MRM transitions was monitored for each period according to the metabolites eluted within the period. Differentially accumulated metabolites were identified in each sample pair [fold-change ≥ 2 or ≤ 0.5, variable importance ≥ 1].
